# Rice *EARLY SENESCENCE 2*, encoding an inositol polyphosphate kinase, is involved in leaf senescence

**DOI:** 10.1186/s12870-020-02610-1

**Published:** 2020-08-26

**Authors:** Shenglong Yang, Guonan Fang, Anpeng Zhang, Banpu Ruan, Hongzhen Jiang, Shilin Ding, Chaolei Liu, Yu Zhang, Noushin Jaha, Peng Hu, Zhengjin Xu, Zhenyu Gao, Jiayu Wang, Qian Qian

**Affiliations:** 1 Key Laboratory of Northeast Rice Biology and Breeding, Ministry of Agriculture/Rice Research Institute, Shenyang Agricultural University, Shenyang, 110866 People’s Republic of China; 2 State Key Laboratory of Rice Biology, China National Rice Research Institute, Hangzhou, Zhejiang 310006 People’s Republic of China

**Keywords:** Early senescence, *es2*, *OsIPK2*, Inositol polyphosphate kinase, Rice (*Oryza sativa L.*)

## Abstract

**Background:**

Early leaf senescence influences yield and yield quality by affecting plant growth and development. A series of leaf senescence-associated molecular mechanisms have been reported in rice. However, the complex genetic regulatory networks that control leaf senescence need to be elucidated.

**Results:**

In this study, an *early senescence 2* (*es2*) mutant was obtained from ethyl methanesulfonate mutagenesis (EMS)-induced mutational library for the *Japonica* rice cultivar Wuyugeng 7 (WYG7). Leaves of *es2* showed early senescence at the seedling stage and became severe at the tillering stage. The contents of reactive oxygen species (ROS) significantly increased, while chlorophyll content, photosynthetic rate, catalase (CAT) activity significantly decreased in the *es2* mutant. Moreover, genes which related to senescence, ROS and chlorophyll degradation were up-regulated, while those associated with photosynthesis and chlorophyll synthesis were down-regulated in *es2* mutant compared to WYG7. The *ES2* gene, which encodes an inositol polyphosphate kinase (OsIPK2), was fine mapped to a 116.73-kb region on chromosome 2. DNA sequencing of *ES2* in the mutant revealed a missense mutation, ES2 was localized to nucleus and plasma membrane of cells, and expressed in various tissues of rice. Complementation test and overexpression experiment confirmed that *ES2* completely restored the normal phenotype, with chlorophyll contents and photosynthetic rate increased comparable with the wild type. These results reveal the new role of *OsIPK2* in regulating leaf senescence in rice and therefore will provide additional genetic evidence on the molecular mechanisms controlling early leaf senescence.

**Conclusions:**

The *ES2* gene, encoding an inositol polyphosphate kinase localized in the nucleus and plasma membrane of cells, is essential for leaf senescence in rice. Further study of *ES2* will facilitate the dissection of the genetic mechanisms underlying early leaf senescence and plant growth.

## Background

Leaf senescence is an internally programmed degeneration phase in the life span of leaf. During growth and development, plants have both stress-induced and age-related senescence. From seedling to maturity, leaves are the main source of photosynthesis. At maturity stage, leaf cells undergo drastic physiological and metabolic changes in an orderly manner, such as chlorophyll degradation, oxidation and hydrolysis of proteins, lipids and nucleic acids [1]. These hydrolyzed metabolites remobilize to new bud, stem or root in perennial plants or to seed in annual plants [2]. In case of early leaf senescence, chloroplasts are the first organelles to be degraded. As a consequence, chlorophyll pigments are disrupted, leaf color changes gradually from green to yellow, brown and finally wither [3]. Simultaneously, decreased photosynthesis and lower assimilates accumulation in reproductive organs are regulated by genetic factors and often triggered by environmental stress which leads to yield losses [4–6]. In some hybrid rice varieties, early senescence leads to impaired leaf function and reduced accumulation of photosynthetic products, which ultimately impact rice yield [7, 8]. As leaves with delayed senescence stayed photosynthetically active and the flowering period extended, plants were shown to set more seeds and accumulate more biomass than the wild type [9]. Therefore, it is important to understand the mechanism underlying early leaf senescence for rice breeding.

The most common physiological alteration during early senescence includes chlorophyll degradation, reactive oxygen species (ROS) scavenging, carbon and nitrogen imbalances, and hormone responses as a coordinated action at the cellular, tissue, organ and organism levels [10]. In the last few decades, series of genes associated with leaf senescence have been isolated and characterized in rice, including transcription factors, receptors and signaling components of hormones or stress responses, and metabolic regulators [4, 11–18]. According to the metabolic pathway involved, senescence genes can be classified into five categories. The first type are also involved in chlorophyll degradation, such as the mutation of *RLS1* and *Osh69* causes, chlorophyll degradation and accelerate leaf senescence [19, 20]. In addition, functional and non-functional stagnant green type gene that hinders the degradation of chlorophyll in late growth, including *NON-YELLOW COLORING 1* (*NYC1*), *NON-YELLOW COLORING 3* (*NYC3)*, *NYC1-LIKE* (*NOL*) and *STAY GREEN RICE* (*SGR*) [21–24]. The second type of senescence-related genes are associated with phytohormones and transcription factors that regulate leaf senescence. For example, abscisic acid (ABA) induces OsNAP to regulate chlorophyll degradation, which affects nutrient transport and expression of senescence-related genes [25]. *OsFBK12* was reported to be involved in ethylene (ETH) signaling pathways promoting leaf senescence [26]. *ZOG1* gene encodes zeatin glucosyltransferase, a Cytokinin (CTK) synthesis-related gene, and mediates senescence of flag leaf in rice [27]. In *Arabidopsis, SAUR* is an Indole-3-acetic acid (IAA) response factor. In order to study the cellular function of plant *SAUR*, knock-out gene was utilized to generate mutants with non-expression in *Arabidopsis*, and resulted to delayed leaf senescence phenotype [28]. The third type of genes related to senescence, which are involve in energy metabolism pathway, including *OsNaPRT1*/*LTS1* gene. Mutation of *LTS1* led to reduction of NAD content, inhibiting the deacetylation ability of OsSRTs*.* This activates senescence-related genes by increasing the acetylation of H3K9, which ultimately lead to senescence of rice leaves [12]. The fourth type are nitrogen remobilization related, such as *Osl2* and *OsFd-GOGAT* [29, 30]. Other genes, such as *SPOTTED LEAF 29* (*SPL29*), *OsSWEET5*, *SENESCENCE 1* (*ES1/TUTOU1*), *OsGDCH* and *DWARF AND EARLY-SENESCENCE 1* (*DEL1*) [3, 31–34]. Most of early senescence mutants exhibited defects in plant growth and development, such as dwarfism or semi-dwarf, withered leaf tip and early heading date [3, 12, 17, 35]. Therefore, identification of premature senescence genes is of great significance to explore the mechanism for early senescence and improve rice yield.

Inositol polyphosphates are important class of organic phosphorus compounds and signaling molecules, which are widely distributed in diverse organisms [36, 37]. Inositol polyphosphate kinase (IPMK/IPK2) is a key enzyme in inositol phosphate metabolism, which converts inositol 1,4,5-trisphosphate (IP3) to inositol tetrakisphosphate (IP4) and inositol pentakisphosphate (IP5) [38–40]. In eukaryotic cells, IPK2-mediated production of IP4 and IP5 for cellular growth, yeast strain bearing deletion of ScIPK2 displays a temperature-sensitive growth defect [41]. ScIPK2 is also an indispensable component of the ArgR-Mcm1 transcriptional complex, which regulates arginine metabolism [42]. In eukaryotes, they are involved in multiple biological functions, such as programmed cell death, hormone signal transduction, ion channels regulation and sensitivity to oxidative stress [37, 43–46]. For instance, the auxin biosynthesis, transport and mediation of axillary shoot branching were also regulated by inositol polyphosphate kinase gene (*AtIPK2α* and *AtIpk2β*) [47]. The overexpression of *AtIPK2α* enhanced root growth through the regulation of inositol trisphosphate (IP3)-mediated calcium accumulation, and overexpression of *AtIpk2β* led to more axillary shoot branches in *Arabidopsis* [47, 48]. *AtIPK2β* is participated in the synthesis of myo-inositol 1,2,3,4,5,6-hexakisphosphate (IP6, phytate). *AtIPK2b* interacts with sucrose non-fermenting-1-related protein kinase (SnRK1.1), which is involved in glucose suppression of seed germination, vegetative growth, flowering and senescence [49–53]. Inositol polyphosphate kinase gene (*OsIPK2*) has been previously isolated and identified as a candidate phytic acid biosynthetic gene in rice. Up-regulation of *OsIPK2* in anthers suggests that a phospholipase C (PLC)-mediated pathway is active in addition to a lipid-independent pathway in anthers [54]. The inositol polyphosphate multikinase (IPMK) acts as a transcriptional activator that binds to the tumor suppressor P53 in mammals, thereby promoting p53-mediated cell death [55]. Although the function of *IPK2* has been reported in mammals and plants, no genetic study has been published on its role in early leaf senescence of rice.

To study molecular mechanism underlying leaf senescence in rice, a new leaf senescence mutant, *early senescence 2* (*es2*), was isolated from ethyl methanesulfonate (EMS)-treated *Oryza sativa japonica cv*. Wuyugeng 7 (WYG7). From the seedling stage, yellow spots appeared in leaves and gradually withered until the tillering stage. The *es2* mutant showed lower chlorophyll content, photosynthetic rate and higher ROS compared to WYG7. Map-based cloning and sequence analysis of the *ES2* gene revealed that it encodes inositol polyphosphate kinase (OsIPK2). Complementation test and overexpression analysis demonstrated that *ES2*/*OsIPK2* mutations causes leaf early senescence, and influences yield-related traits in rice.

## Results

### Early leaf senescence and decreased agronomic traits in the *es2* mutant

**Fig. 1:**
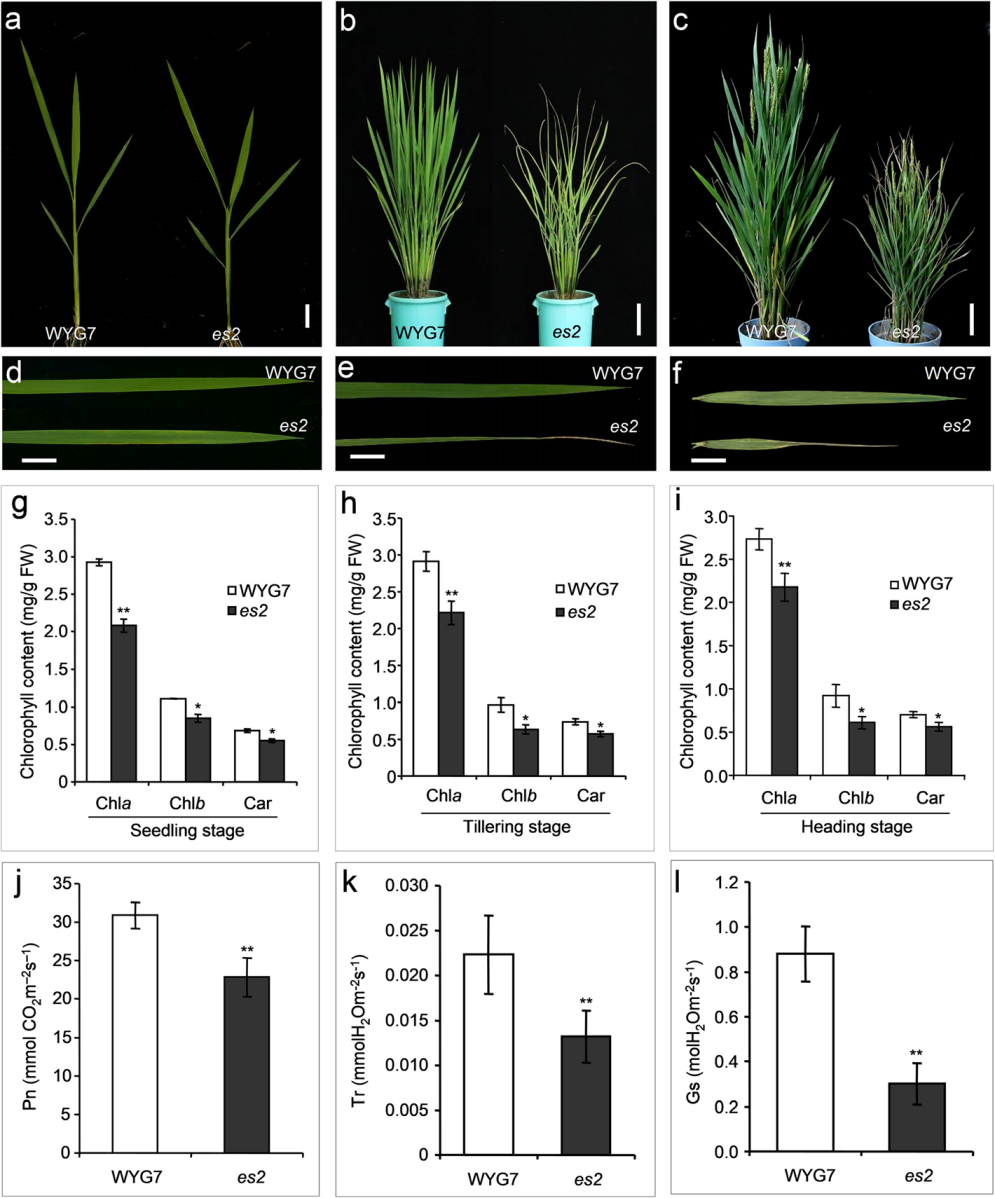
Comparison of phenotypic and physiological characteristics between the *es2* mutant and the wild-type WYG7. **a**-**c** Phenotype between *es2* and the wild-type WYG7 at seedling stage (Scale bar = 2 cm), tillering stage (Scale bar = 10 cm), and heading stage (Scale bar = 10 cm), respectively. **d**-**f** Close-up image of the leaves in WYG7 and *es2* at seedling stage (Scale bar = 2 cm), tillering stage (Scale bar = 2 cm), and heading stage (flag leaf, Scale bar = 2 cm), respectively. **g**-**i** Contents of chlorophyll *a*, *b* and Car at seedling, tillering and heading stage, respectively. **j-l** Net photosynthetic rate (Pn), transpiration rate (Tr) and Stomatal conductance (Gs) at tillering stage. Mean ± SD, *n* = 3, * significance at *P* < 5%, ** extremely significance at *P* < 1% (Student’s *t*-test)

We identified a mutant, *es2*, with an early leaf senescence phenotype from the EMS mutagenesis mutant population of *Japonica* variety, WYG7. The *es2* mutant showed leaf senescence from the seedling stage until the maturity stage (45 days after pollination). At the seedling stage, yellow spots distributed throughout the leaves, and the aging leaves gradually withered (Fig. 1a-f). In order to analyze the leaf senescence phenotype more precisely, the detached leaves of WYG7 and the *es2* mutant at 2-leaf stage were kept in the dark at 28 °C. Compared to WYG7, the leaves of *es2* almost turned more yellower after 5 days in darkness. This indicates that mutation in *ES2* accelerates dark-induced leaf senescence (Additional file 5: Figure S1).

In addition, the *es2* mutant showed significant decrease in some agronomic traits including plant height, internode length, panicle length, number of primary branches, number of secondary branches, grain width and 1000-grain weight compared to wild-type plants. Moreover, the number of tillers increased significantly compared to the wild-type plants (Additional file 6: Figure S2). These results indicates that the early senescent leaves in *es2* negatively affects yield.

### Decreased chlorophyll content and photosynthetic ability with abnormal chloroplast ultrastructure in the *es2* mutant

**Fig. 2:**
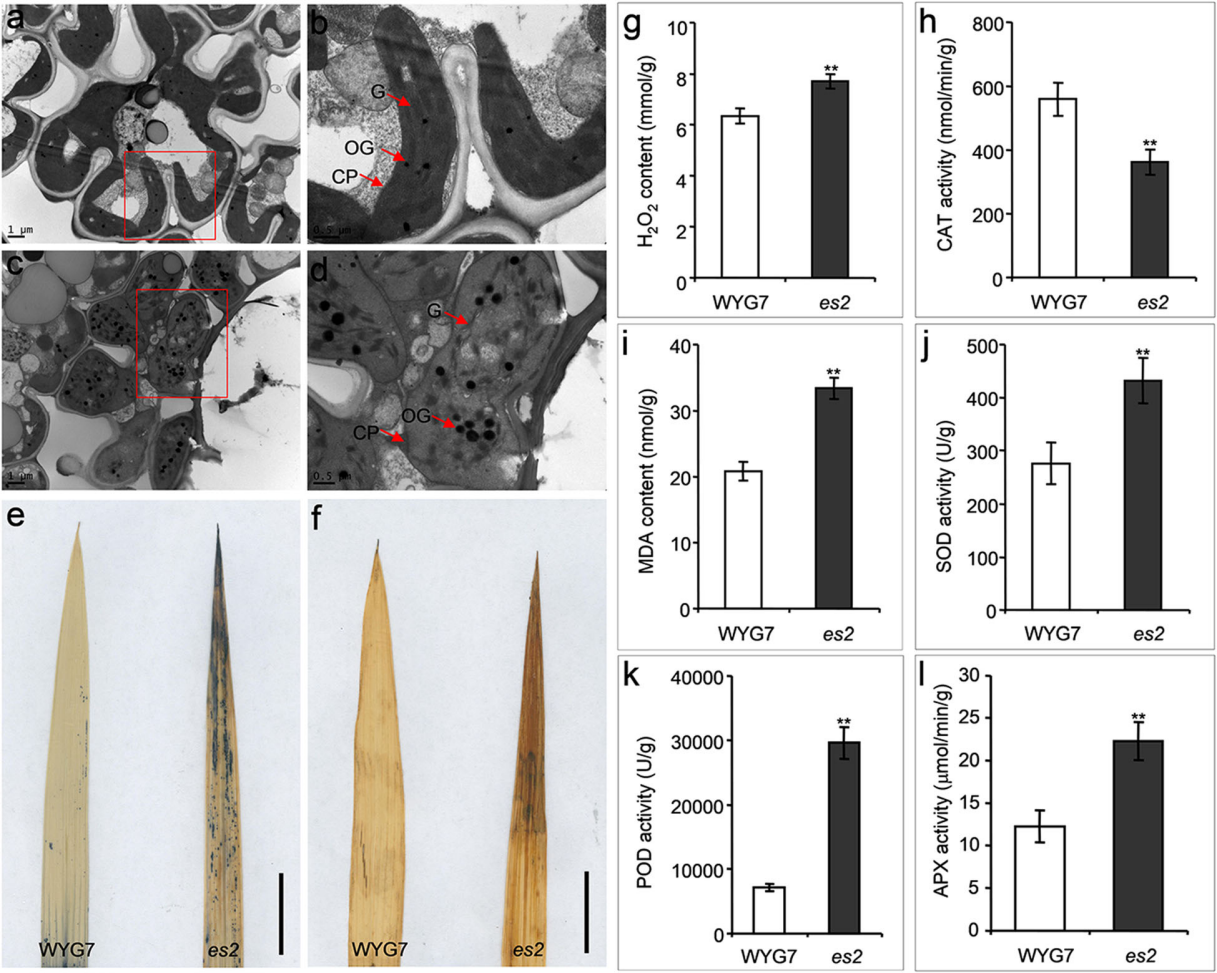
Ultrastructure of the chloroplast, DAB and NBT staining, and senescence related-indices determination in WYG7 and *es2* at the tillering stage. **a**, **b** Ultrastructure of the chloroplast at the tillering stage of WYG7. **a** Scale bar = 1 μm, (**b**) Scale bar = 0.5 μm. **c**, **d** Ultrastructure of the chloroplast at the tillering stage of *es2*. **c** Scale bar = 1 μm, (**d**) Scale bar = 0.5 μm. CP, chloroplast; G, granum; OG, osmiophilic granule. **e** NBT straining. Scale bar = 2 cm. **f** DAB straining. Scale bar = 2 cm. **g** H_2_O_2_ content. **h** Catalase (CAT) activity. **i** malondialdehyde (MDA) content. **j** Superoxide dismutase (SOD) activity. **k** Peroxidase (POD) activity. **l** ascorbate peroxidase (APX) activity. Mean ± SD, *n* = 3. * significance at *P* < 5%, ** extremely significance at *P* < 1% (Student’s *t*-test)

Compared to WYG7, chlorophyll *a*, *b*, and Carotenoid (Car) contents significantly decreased in the *es2* mutant at seedling, tillering and heading stages (Fig. 1g-i**)**. Photosynthetic parameters were examined at the tillering stage in WYG7 and the *es2* mutant. Net photosynthetic rate (*P*_n_), transpiration rate (*T*_r_) and stomatal conductance (*G*_s_) were significantly declined respectively in the *es2* mutant compared to the wild type (Fig. 1j-l). Transmission electron microscopy (TEM) analysis showed that the number of chloroplasts dramatically reduced in *es2* mutant compared with in WYG7. The thylakoids and stroma lamellae structure in leaves were disorderly arranged in the *es2* mutant. Simultaneously, more osmiophilic granules (OG) were found in *es2* compared to WYG7. Therefore, mutation in *ES2* led to an abnormal chloroplast development (Fig. 2a-d).

### The *es2* mutant exhibited cell death and more ROS accumulation in leaves

**Fig. 3:**
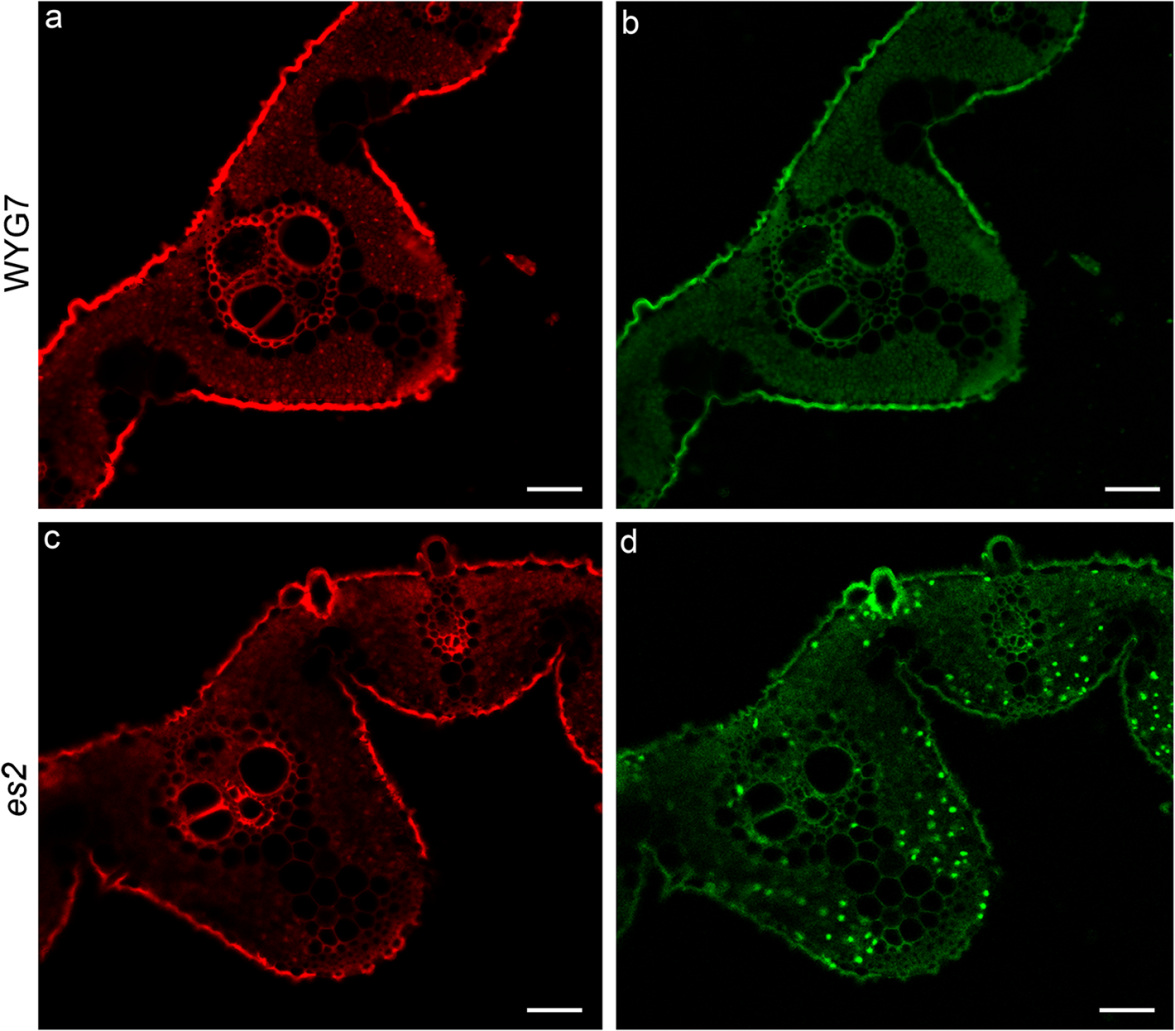
Cell death in WYG7 and *es2* leaves at the tillering stage by TUNEL assay. **a**-**d** TUNEL assay of WYG7 (**a**, **b**) and *es2* (**c**, **d**). Red signal is Propidium Iodide (PI) staining, green fluorescence represents DNA fragments. Scale bar = 50 μm

To determine whether early senescence in *es2* resulted from concomitant accumulation of superoxide radicals, nitroblue tetrazolium (NBT) staining was performed and more blue formazan precipitates were found in *es2* leaves than in WYG7 at the tillering stage (Fig. 2e). We also examined hydrogen peroxide (H_2_O_2_) levels by 3, 3-diaminobenzidine (DAB) staining in both WYG7 and *es2*. The formation of reddish-brown formazan precipitates in the *es2* leaves at the tillering stage indicated the accumulation of H_2_O_2_ (Fig. 2f). Furthermore, the contents of H_2_O_2_ and malondialdehyde (MDA), activities of catalase (CAT), peroxidase (POD), superoxide dismutase (SOD) and ascorbate peroxidase (APX) were measured at the tillering stage in WYG7 and *es2*, respectively. The results showed that H_2_O_2_ and MDA contents were significantly higher in *es2* than in WYG7 (Fig. 2g, i). The activities of POD, SOD and APX increased, while CAT activity decreased in the *es2* mutant. Therefore, the *es2* mutant exhibited more ROS accumulation than the wild type (Fig. 2h, j-l). In order to examine cell death in *es2*, leaves of the *es2* mutant and WYG7 at the tillering stage were tested for TUNEL assay. Stronger green fluorescence was observed in *es2* mesophyll cells than that of WYG7, indicating that a large number of DNA fragments accumulated and cell apoptosis occurred in *es2* (Fig. 3a-d).

### Expression alteration of genes related to senescence, ROS, chlorophyll synthesis and degradation, photosynthesis and chloroplast development in the *es2* mutant

**Fig. 4:**
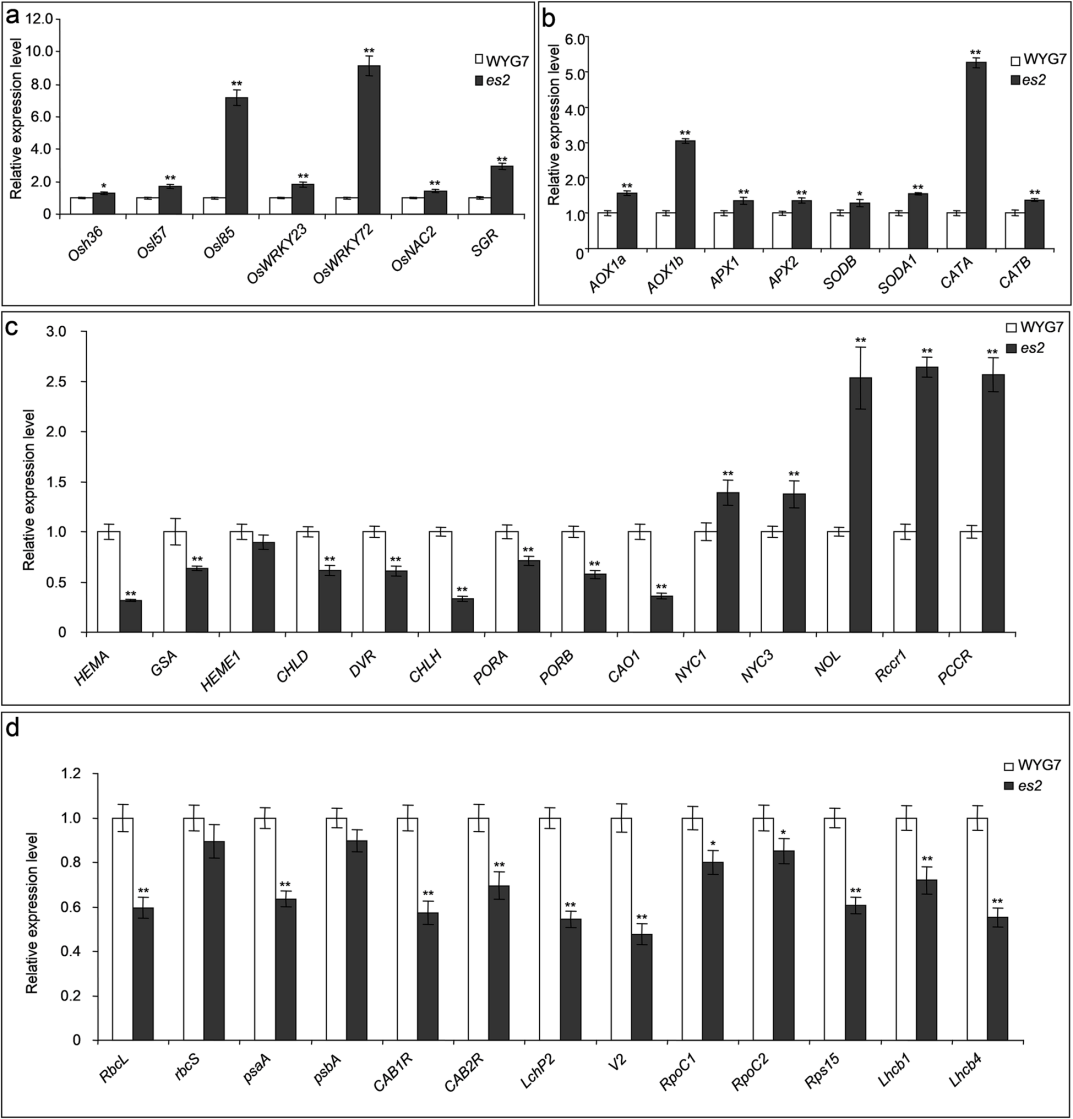
Alteration in expression level of senescence-, ROS-, chlorophyll-, photosynthesis- and chloroplast development-related genes in *es2*. **a** Relative expression levels of senescence-related genes. **b** Relative expression levels of reactive oxygen scavenging system (ROS) related genes. **c** Relative expression levels of chlorophyll synthesis and degradation related genes. **d** Relative expression levels of photosynthesis and chloroplast development related genes. A *Histone* gene was used as the reference. Mean ± SD, n = 3. * significance at *P* < 5%, ** extremely significance at *P* < 1% (Student’s *t*-test)

To understand the molecular basis of early senescence *es2*, we selected genes which were related to senescence, ROS, chlorophyll synthesis and degradation, photosynthesis, and chloroplast development for qRT-PCR analysis. Consistently, the expression levels of several senescence related genes, such as *Osh36*, *Osl57*, *Osl85*, *OsWRKY23*, *OsWRKY72*, *OsNAC2* and *SGR* [24, 56–58] were significantly up-regulated in *es2* at tillering stage in contrast with WYG7 (Fig. 4a). The transcript amounts of ROS associated genes, including *AOX1a*, *AOX1b*, *APX1*, *APX2*, *SODB*, *SODA1*, *CATA* and *CATB* [59–61] were remarkably up-regulated in *es2* (Fig. 4b). qRT-PCR results of genes which were related to synthesis and degradation of chlorophyll showed that except for *HEME1*, the expression levels of *HEMA*, *GSA*, *CHLD*, *DVR*, *CHLH*, *PORA*, *PORB* and *CAO1* [62–67] were significantly down-regulated, while those of *NYC1*, *NYC3*, *NOL*, *Rccr1* and *PCCR* [21–23, 68] were significantly up-regulated in *es2* (Fig. 4c). Except for two genes *rbcS* and *RpoC2*, the expression levels of all tested photosynthesis and chloroplast development related genes, involving *RbcL*, *psaA*, *psbA*, *CAB1R*, *CAB2R*, *LchP2*, *V2*, *RpoC1*, *Rps15*, *Lhcb1* and *Lhcb4* [69–71] were significantly down-regulated in *es2* (Fig. 4d). These results are correlated with the accumulation of ROS, the decrease in photosynthetic capacity and contents of chlorophyll in the *es2* mutant at transcript level.

### Genetic analysis and fine mapping of the *ES2* gene

For genetic analysis of the *es2* mutant, we crossed the mutant with *japonica* variety WYG7. All F_1_ plants showed the wild-type phenotype, suggesting that the mutation is recessive. Among 784 randomly selected F_2_ plants, the number of wild-type plants and the mutant plants were 604 and 180, respectively, consistent with the ratio of 3:1 (χ^2^ = 1.7361). Therefore, the mutant was controlled by a recessive nuclear gene, designated as *ES2* gene (Additional file 1: Table S1).

**Fig. 5:**
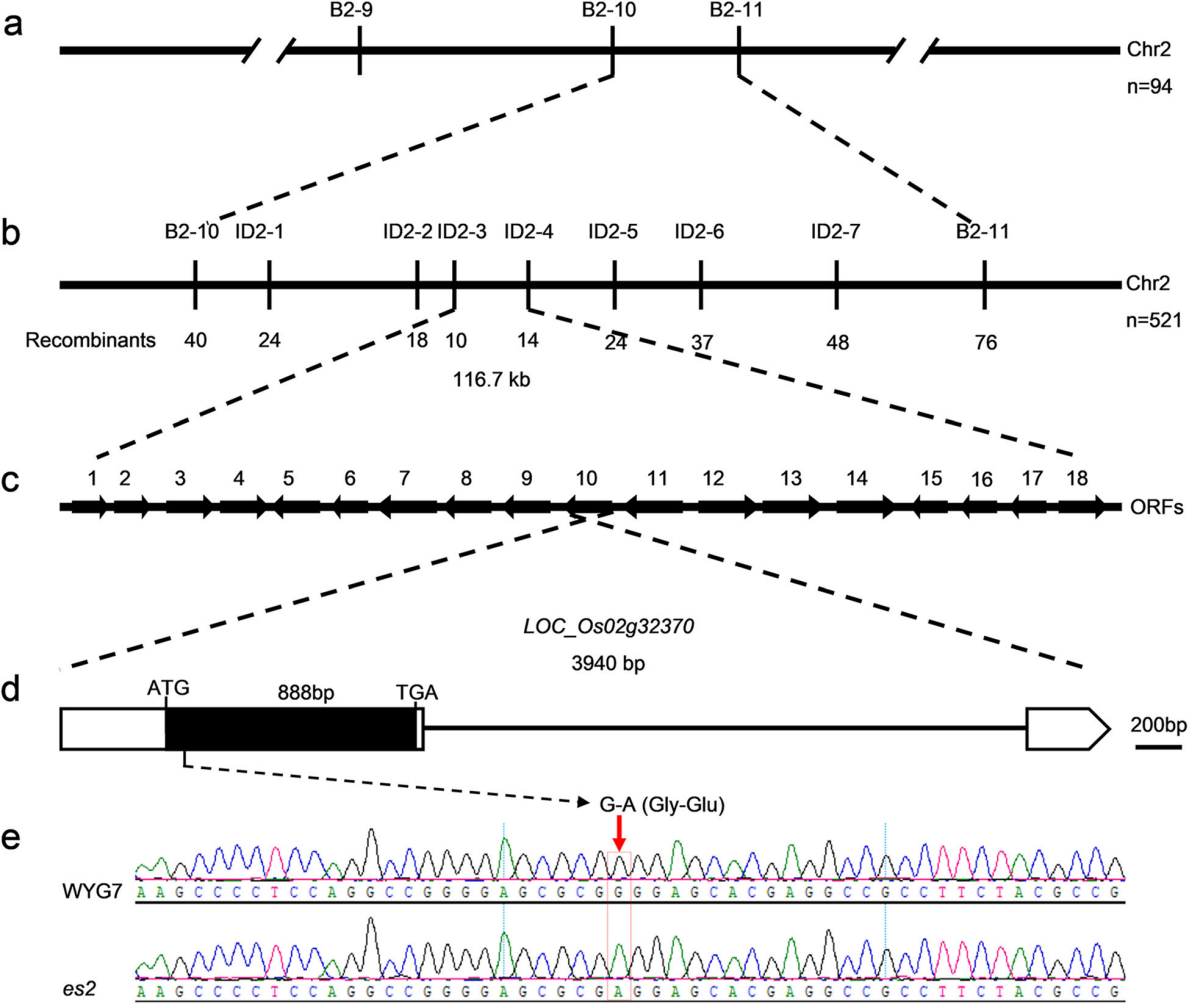
Map-based cloning of *ES2* and identification of mutated site in *es2*. **a** *ES2* was preliminarily mapped between markers B2–10 and B2–11 on chromosome 2. **b** Fine mapping of *ES2*. The *ES2* locus was fine mapped to a 116.7 kb region between markers ID2–3 and ID2–4. **c** Eighteen putative ORFs are located in the 116.7 kb region. **d** Gene structure of the candidate gene *LOC_Os02g32370*. Scale bar =200 bp. The black rectangle represents the exon. The point mutation of G to A on the exon led to amino acid replacement of Gly with Glu. **e** Comparison of sequence chromatograms around the mutated site between the wild-type WYG7 and *es2*

In order to fine map the *ES2* gene, an F_2_ segregating population was derived from the crossing between the *es2* mutant and *indica* variety 93–11. Chromosomal linkage analysis was performed using 94 mutant plants with 225 polymorphic SSR markers distributed on 12 chromosomes. Three SSR/STS markers on chromosome 2, B2–9, B2–10 and B2–11 were linked to *ES2* (Fig. 5a). Total of 94 mutant plants were tested using these 3 markers and the gene was initially mapped between B2–10 and B2–11. To fine map *ES2*, the mapping population was expanded to 521 F_2_ mutant individuals. Based on the comparison between genomic sequences of Nipponbare and 93–11, 7 polymorphic Indel markers were developed between B2–10 and B2–11. Using these markers, the *ES2* gene was fine mapped between ID2–3 and ID2–4 within a 116.73 kb region (Fig. 5b). According to Nipponbare genomic sequence in the region (https://rapdb.dna.affrc.go.jp/), there were 18 annotated ORFs, among which a G to A substitution in exon of the gene *LOC_Os02g32370* in *es2* was detected, which caused replacement of glycine with glutamic acid (Fig. 5c-e).

### Sequence and phylogenetic analysis of the *OsIPK2* gene

**Fig. 6:**
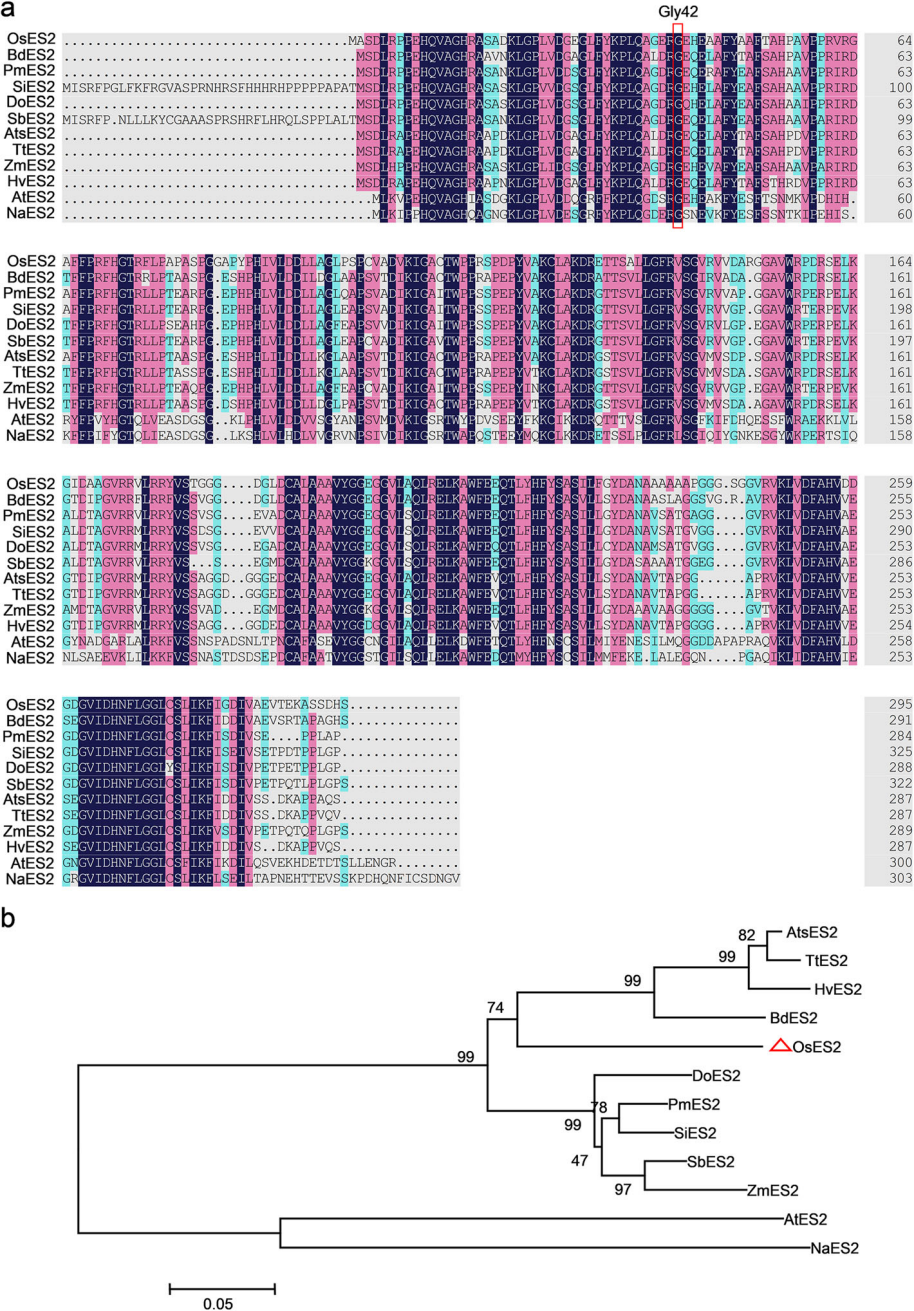
Sequence alignment and phylogenetic analysis of ES2. **a** Alignment of ES2 protein homolgs from 12 plant species. Black or pink shades indicate fully or partially conserved amino acid. **b** Phylogenetic tree of ES2. Protein sequences include *Oryza sativa Japonica* Group (OsES2, XP_015623153.1), *Panicum miliaceum* (PmES2, RLM78467.1), *Sorghum bicolor* (SbES2, XP_002452184.1), *Brachypodium distachyon* (BdES2, XP_003575046.1), *Setaria italica* (SiES2, XP_004952603.2), *Dichanthelium oligosanthes* (DoES2, OEL29654.1), *Aegilops tauschii subsp. tauschii* (AtsES2, XP_020147020.1), *Hordeum vulgare subsp. vulgare* (HvES2, BAJ86494.1), *Zea mays* (ZmES2, PWZ22907.1), *Triticum turgidum subsp. durum* (TtES2, VAI91267.1), *Arabidopsis thaliana* (AtES2, NP_001331991.1) and *Nicotiana attenuate* (NaES2, XP_019247260.1)

The *ES2* gene encodes an inositol polyphosphate kinase (*OsIPK2*). Protein sequence alignment showed that Gly_42_ in OsES2 was highly conserved in plants (Fig. 6a). Phylogenetic tree constructed with homolog sequences of the OsES2 protein from different species showed that they can be divided into two groups: monocots and dicots. Among these species, rice ES2 exhibited the highest similarity (76.57%) to the ortholog in *Brachypodium distachyon* (Fig. 6b).

### *OsIPK2/ES2* gene was responsible for leaf early senescence in rice

**Fig. 7:**
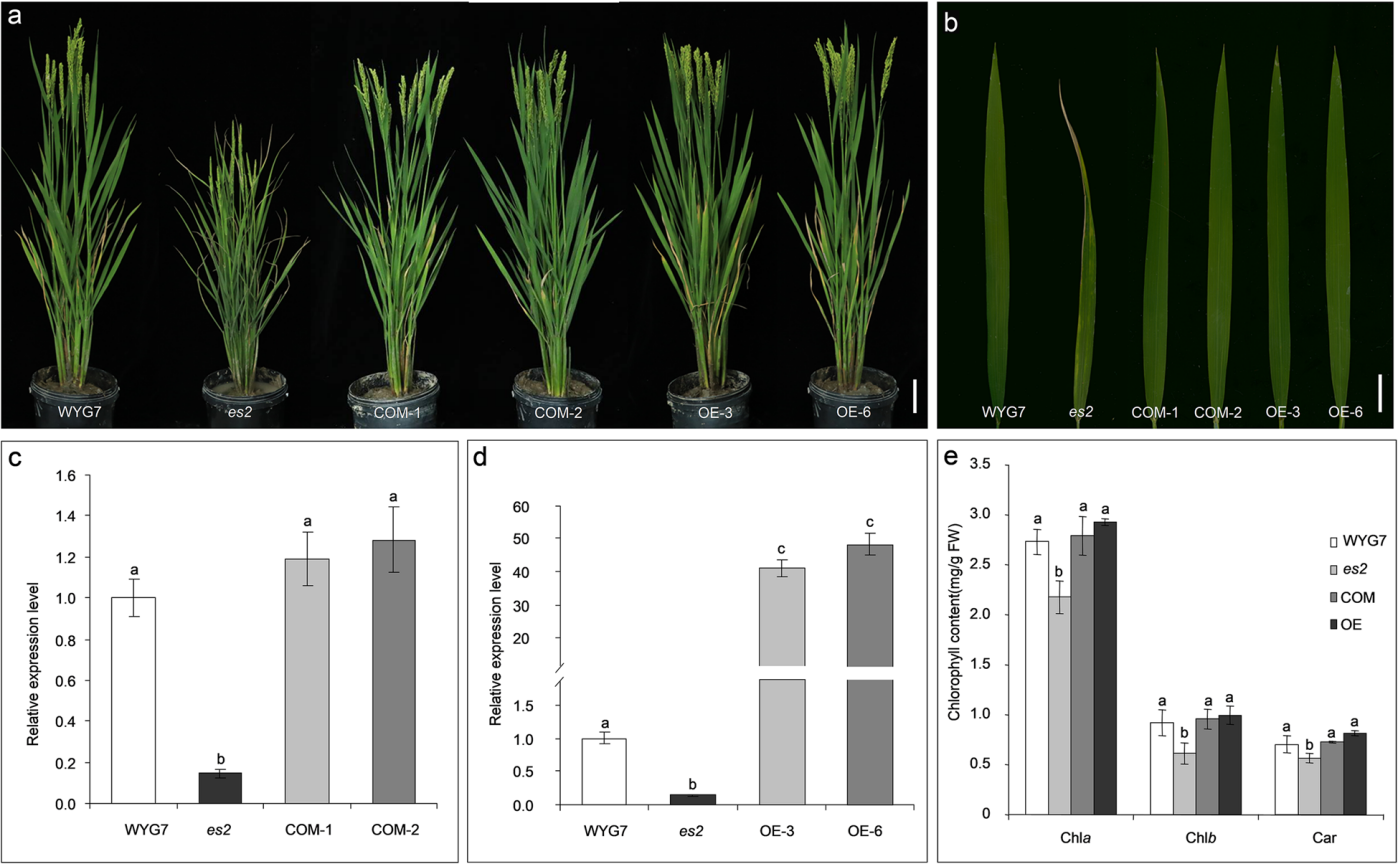
Functional complementation and overexpression of *OsIPK2* restored phenotypes of the *es2* mutant. **a** Plants of WYG7, *es2*, COM-1, COM-2, OE-3 and OE-6 at the heading stage. Scale bar = 10 cm. **b** Close-up image of flag leaves of WYG7, *es2*, COM-1, COM-2, OE-3 and OE-6. Scale bar = 2 cm. **c** Relative expression level of *ES2* in WYG7, *es2* and COM. Mean ± SD, *n* = 3. **d** Relative expression level of *ES2* in WYG7, *es2*, OE-3 and OE-6. Mean ± SD, *n* = 3. **e** Contents of chlorophyll *a*, *b* and Car in flag leaf of WYG7, *es2*, COM-1, COM-2, OE-3 and OE-6 at the heading stage. Mean ± SD, *n* = 3. a, b, c indicate a significant difference at the 1% level (Student’s *t*-test)

In order to confirm *OsIPK2* the *ES2* gene, complementation test was conducted and all of the 13 transgenic plants restored the wild-type phenotype (Fig. 7a, b). As expected, gene expression level, chlorophyll contents and photosynthetic parameters were restored to those of the wild type (Fig. 7c, e; Additional file 7: Figure S3). Furthermore, overexpression experiment showed all the 9 transgenic plants restored the wild-type phenotype (Fig. 7a, b). With dramatic increase in expression levels of *OsIPK2*, chlorophyll contents and photosynthetic parameters were also elevated (Fig. 7d, e, Additional file 7: Figure S3).

### ES2 was localized to nucleus and plasma membrane and expressed in various tissues of rice

To determine subcellular localization of the ES2 protein, the p35S::ES2::GFP vector was transferred into rice protoplasts and tobacco (*N. benthamiana*), respectively, with the p35S::GFP empty vector as control (Additional file 8: Figure S4a-c, g-i). The p35S::ES2::GFP vector was transferred into rice protoplasts, and fluorescence confocal observation revealed that ES2 was localized to nucleus and plasma membrane (Additional file 8: Figure S4d-f). Consistently, fluorescent signals were also observed in nucleus and plasma membrane in epidermal leaf cells of tobacco (*N. benthamiana*) transformed with the p35S::ES2::GFP vector (Additional file 8: Figure S4j-l). Besides, the p35S::ES2::YFP vector was transferred into rice protoplasts, and fluorescence confocal observation revealed that ES2 was also localized to nucleus and plasma membrane (Additional file 9: Figure S5).

The β-glucuronidase (GUS) staining observations detected *ES2* expressed in various tissues, including roots, stems, leaves, sheaths and panicles with strongest in leaves (Additional file 10: Figure S6a-e). Consistently, qRT-PCR results showed that *ES2* were expressed in roots, stems, leaves, sheaths and panicles, with highest expression level in leaves followed by stems, panicles, roots and sheaths (Additional file 10: Figure S6f).

## Discussion

Leaf senescence is an essential developmental stage of programmed procedure, and accompanied by the emergence of characteristics in plants, such as changes in leaf color, degradation of chloroplast, and reduction in chlorophyll content and photosynthetic efficiency. Eventually, early leaf senescence may cause plant growth and development retardation, and yield reduction [72]. Recently, a series of leaf senescence-associated genes have been cloned and characterized in rice [3, 12, 17, 33, 35, 73]. The lower leaves of *premature leaf senescence 3* mutant (*pls3*) firstly turned yellow at tillering stage, and senescence becomes severe at the mature stage. The *pls3* mutant also showed decreased chlorophyll and melatonin contents, shorter plant height, and 1000-grain weight [17]. The tips and margins of the lower leaves of *es4* mutant became yellow at tillering stage, and senesce at the grain-filling stage. The *es4* mutant also showed reduced chlorophyll content and photosynthetic rate, shorter plant height, and 1000-grain weight [73]. In this study, the *es2* mutant also showed rapid leaf senescence, different from previously reported mutants. Firstly, the early senescence of plants is accompanied by changes in chlorophyll content. The leaves color changed from green to yellow spot at seedling stage, and alteration of withered at tillering stage until maturity (Fig. 1a-f), while the degradation of chlorophyll content and lower photosynthetic rate (Fig. 1g-l). Secondly, the plant height, internode length, panicle length and thousand-grain weight were significantly reduced in *es2* mutant (Additional file 6: Figure S2). These results showed that the senescence process in the *es2* mutant began earlier than in previously reported mutants, therefore, *es2* is a novel mutant to dissect the mechanism of leaf senescence.

In higher plants, there were some reports about inositol polyphosphate kinase involved in multiple biological functions. There are two *IPK2* genes in Arabidopsis, *AtIPK2α* and *AtIpk2β*, and only one in rice [54]. In Arabidopsis, *IPK2* plays important role in axillary shoot branching, axillary shoot branching, root growth, synthesis of phytic acid, abiotic stress responses, auxin response, seed germination, vegetative growth, flowering and senescence [47–53, 74, 75]. *OsIPK2* has been previously isolated and identified as a candidate biosynthetic gene for phytic acid in rice [54]. However, their physiological functions have not been reported. Recently, it was reported that inositol polyphosphate kinase directly interacts with *OsIAA11* to regulate lateral root formation and was involved in gibberellic acid signaling modulation to affect shoot elongation and fertility [76, 77]. In this study, different from previously reported genes, the early senescence phenotype was caused by a mutation in the *ES2*/*OsIPK2* gene for inositol polyphosphate kinase. *ES2* expression was detected in all tissues surveyed, but predominantly in leaf mesophyll cells. The qRT-PCR results showed that the expression level of *OsIPK2* in *es2* was significantly up-regulated at seedling stage, while down-regulated at tillering and heading stage compared with WYG7 (Additional file 11: Figure S7). Additionally, the mutated *ES2* gene was transformed into the *es2* mutant plants and could not restore the wild-type phenotype, although expression level of the gene was significantly increased compared with *es2* mutants (Additional file 12: Figure S8). Considering the occurrence of leaf senescence in the *es2* mutant from seedling stage until maturity, phenotypic change caused by a single nucleotide mutation in *ES2*/*OsIPK2* might not be correlated with gene expression level. In order to find whether the mutation impairs kinase activity towards the phytic acid production level, we measured phytic acid content in the wild-type WYG7 and e*s2* mutants. The contents of phytic acid were significantly reduced in e*s2*, indicating that the mutation in *IPK2* hampers phytic acid biosynthesis (Additional file 13: Figure S9).

Previous studies showed that early leaf senescence was associated with the accumulation of ROS [17, 32, 78], especially H_2_O_2_ accumulation [79–81]. ROS accumulation led to oxidative damage in the thylakoid membranes and other cellular components [82]. Here, NBT and DAB staining showed that O_2_^−^ and H_2_O_2_ accumulated in the *es2* mutant compared with the wild-type WYG7 (Fig. 2e, f). We speculate that reduction in chlorophyll content and abnormality in chloroplast ultrastructure in *es2* are due to oxidative damage caused by ROS. The qRT-PCR analysis showed that expression levels of senescence-associated genes were up-regulated in the *es2* mutant (Fig. 4a). TUNEL assay also showed that a large number of DNA fragments appeared in cells of *es2* (Fig. 3). Furthermore, the *ES2* protein localized to the nucleus and plasma membrane (Fig. S3), wherein large amounts of ROS were produced. These results suggests that leaf senescence may lead to by ROS accumulation.

During early senescence, plant leaves undergo a series of physiological changes, such as alteration in contents of H_2_O_2_ and MDA, activities of CAT, SOD, POD and APX, and cell death [73, 83–85]. In early senescence, SOD and CAT can remove ROS [86, 87]. SOD catalyzes O^2−^ dismutase to produce H_2_O_2._ CAT is the major H_2_O_2_-scavenging enzyme. APX also plays an important role in control of H_2_O_2_ level in cells [73, 85, 88]. In our study, activities of SOD, POD and APX in *es2* were significantly higher than those in WYG7, while activity of CAT was significantly lower than that of WYG7 (Fig. 2h, j-l). This is consistent with previous reports concerning early senescence mutants in rice [17, 73, 89–91]. Therefore, it is indicates that the increased SOD activity in *es2* was due to high O_2_^−^ production and reducedCAT activity accelerated accumulation of H_2_O_2_. The increased MDA content in leaves provided further evidence of lipid peroxidation and ROS accumulation in *es2* (Fig. 2i). Leaf senescence is mediated by a large number of ROS related genes, such as *AOX1a*, *AOX1b*, *APX1*, *APX2*, *SODB*, *SODA1*, *CATA* and *CATB*. The qRT-PCR results showed that their expression levels were significantly up-regulated in *es2* compared to WYG7 (Fig. 4b).

## Conclusions

In conclusion, the mutation of *ES2*/*OsIPK2* gene resulted to increased H_2_O_2_ and MDA contentand CAT, SOD, POD and APX activity, and reduced and chlorophyll and *P*_n_ content, which eventually leads to leaf senescence and reduced rice yield.

## Methods

### Plant materials, growth conditions and dark treatment

The *japonica* variety Wuyugeng 7 (WYG7) and *indica* variety 93–11, were conventionally cultivated in China. These seeds were provided by the China National Rice Research Institute (CNRRI) in Hangzhou, Zhejiang Province, China. Early senescence leaf mutant *es2* was obtained by EMS mutagenesis of WYG7. The mutant was crossed with *indica* variety 93–11, which was used to construct F_2_ mapping population. The *es2* mutant and progenies showed stable inheritance and exhibited premature senescence. All rice plants were grown in the fields of CNRRI in Hangzhou, Zhejiang Province, China. For the dark treatment, the detached leaves from 2-leaf stage plants (before yellow spots appearing) grown in an incubator were incubated on ddH_2_O at 28 °C in complete darkness for 5 days.

### Detection of chlorophyll content

The fresh leaves from wild-type plants and the *es2* mutant were sampled in different growth periods in field conditions. The fresh leaves were cut into small pieces of about 0.5 cm length and 0.05 g weight after removing the main leaf veins, then placed into 5.0 ml 80% acetone and soaked for 24 h in the dark, with shaking every 8 to 10 h until the photosynthetic pigment was fully dissolved. The 1 ml sample solution was measured at 470 nm, 645 nm and 663 nm using visible spectrophotometer (BACKMAN COULTER DU800, United States). The experiment was performed with three biological replicates per group, and *t*-test was conducted in statistical analysis. The formulas used to calculate chlorophyll *a* (Chl *a*), chlorophyll *b* (Chl *b*) and carotenoid (Car) contents are listed as following [17]:

$$ {\displaystyle \begin{array}{c}\mathrm{Chl}a=\left(12.7\times A663-2.69\times A645\right)\times V/W;\\ {}\mathrm{Chl}b=\left(22.9\times A645-4.68\times A663\right)\times V/W;\\ {}\mathrm{Car}=\left(1000\times A470\times V/W-3.27\times \mathrm{Chl}a-104\times \mathrm{Chl}b\right)/198.\end{array}} $$

### Photosynthetic rate measurement

In case of sunshine day at 9:00, the net photosynthetic rate (*P*_n_), transpiration rate (*T*_r_), and stomatal conductance (*G*_s_) of fully expanded leaves from WYG7 and the *es2* mutant were measured by portable photosynthesis measurement device LI-6400 (Li-Cor, Lincoln, NE, United States) with 28 °C, 1200 μmol photons m^− 2^ s^− 1^ irradiance and 400 μmol mol^− 1^ CO_2_ concentration under field conditions at tillering stage. Three biological replicates were used and *t*-test was conducted for statistical analysis.

### Transmission electron microscopy analysis of chloroplast ultrastructure

Fresh leaves of the wild-type WYG7 and the *es2* plants at the seedling stage and the tillering stage in field were sampled, and the main vein was removed. A small section cut into about 0.5–1 cm segments and immediately placed in 2 ml containing 2.5% glutaraldehyde fixative, air was removed by a vacuum for about 2 h until completely sinked to the bottom of the tube and kept at 4 °C for more than 16 h. The segments were washed 3 times in the phosphate buffer (0.1 M, PH 7.0) for 15 min at each step, and treated with 1% (w/v) OsO_4_ in phosphate buffer for 1–2 h. After washing 3 times in the phosphate buffer for 15 min at each step, the samples were dehydrated using a gradient of ethanol solutions from 30 to 100% for 15 min at each step. The samples were placed in 1:1 mixture of alcohol and 90% acetone for 20 min at room temperature. Next, the samples were transferred into 90% acetone for 20 min and then into 100% acetone for dehydration treatment 3 times for 15 min at each step. After dehydration treatment, the samples were transferred into a final Spurr resin mixture overnight. The specimens were then placed in capsules with embedding medium and heated at 70 °C for 9 h. The specimen sections were stained using uranyl acetate and alkaline lead citrate for 15 min each, then observed by electron microscopy (Hitachi H^− 7650^, Tokyo, Japan).

### Nitro blue tetrazolium (NBT) and 3, 3′-diaminobenzidine (DAB) staining

Qualitative analysis of superoxide anion (O_2_^−^) and hydrogen peroxide (H_2_O_2_) were carried by nitroblue tetrazolium (NBT) and 3, 3′-diaminobenzidine (DAB) staining [92, 93]. Fresh leaves of WYG7 and *es2* were obtained from plants at tillering stage in paddy fields, and the samples were incubated in 0.05% (w/v) NBT or 0.1% (w/v) DAB (pH 5.8) with gentle shaking at 28 °C in dark for 12 h.

### Determination of senescence-related physiological index and phytic acid

The contents of hydrogen peroxide (H_2_O_2_) and malondialdehyde (MDA), activities of catalase (CAT), superoxide dismutase (SOD), peroxidase (POD) and ascorbate peroxidase (APX) were measured using an Assay Kit (Suzhou Keming Biotechnology Co, Ltd. In). The leaves of WYG7 and *es2* were sampled from plants grown in the paddy field at tillering stage. The contents of phytic acid were measured using an Assay Kit (Suzhou Keming Biotechnology Co, Ltd. In). The leaves of WYG7 and *es2* were sampled from plants grown in the paddy field at seedling, tillering and heading stage. Three biological replicates were used and *t*-test was conducted.

### Detection of apoptosis by TUNEL assay

The leaves were fixed with FAA fixative and embedded in paraffin at the tillering stage. The sections were microscopically examined to select a suitable slide and dewaxed by gradient alcohol. TUNEL staining was performed according to Huang et al. (2001) [94], and apoptosis was detected by DeadEnd™ Fluorometric TUNEL system kit (Promega, Wisconsin, USA). The localized green fluorescence (520 nm) of apoptotic cells (fluorescein-12-dUTP) in a red (620 nm) background (Propidium Iodide, PI) was detected by laser-scanning confocal microscope (Zeiss LSM700, Carl Zeiss, Inc., Thornwood, NY, USA).

### Genetic analysis and fine mapping

A reciprocal cross between *es2* and the *japonica* WYG7 was performed for genetic analysis. The F_2_ segregating populations were used for the χ^2^ test. The genetic analysis information used was listed in Additional file 1: Table S1. For fine mapping, F_2_ segregating populations were derived from the cross between *es2* mutant and the *indica* 93–11. F_2_ plants were grown in the paddy fields in Hangzhou, which were used for segregation analysis. 94 F_2_ individual plants phenotyped as mutants were used for the initial localization of *es2*. 521 F_2_ mutant individual plants were used for fine mapping. Total DNA samples were extracted from the leaves using the cetyltriethyl ammonium bromide (CTAB) method. The initial mapping was conducted by 225 SSR and Indel markers, which distributed across 12 chromosomes in rice (http://www.Gramene.org). For fine mapping of the *ES2* gene, the new Indel markers were designed by Primer Premier 5.0 after comparing the sequence between Nipponbare and 93–11 in Gramene website. The marker information used were listed in Additional file 2: Table S2.

### Sequence and phylogenetic analysis

The ES2 protein sequence composed of 295 amino acids was obtained from Gramene website (http://www.gramene.org/). A homologous analysis of ES2 was performed using the BLASTP program on National Center for Biotechnology Information website (NCBI, https://www.ncbi.nlm.nih.gov/). DNAMAN was used to aligned sequences. MEGA 7.0 software with the bootstrap method and 1000 replicates was used to construct a phylogenetic tree of *ES2* and homologous proteins.

### Plasmid construction and plant transformation

To verify whether *LOC_Os02g32370* was the *ES2* gene, a 6030 bp genome sequence that includes the *ES2* coding region along with the upstream sequence and downstream sequences was amplified from wild-type WYG7 by PCR using the ES2-COM primer, and inserted into pCAMBIA1300 vector. In order to construct an overexpression vector, 888 bp CDS was inserted into pCAMIA1300s vector. All vectors were transformed into the *es2* mutants by *Agrobacterium* (EHA105)-mediated transformation. Sequences of primers used were listed in Additional file 3: Table S3.

### Subcellular localization and GUS assay

Primers with Sal *I* linker were designed to amplify the CDS sequence of WYG7, which was ligated to GFP vector pCAMBIA1301–35S-S65T-GFP by recombinant method, transformed into rice protoplasts [95], and transformed into tobacco (*N. benthamiana*) leaf epidermal cells with the protocol described by Ruan et al. (2019) [96]. Besides, YFP vector pCAMBIA1300-35S-YFP by recombinant method was also transformed into rice protoplasts. The transient expression of *es2* was analyzed. GFP and YFP signals were observed by laser-scanning confocal microscopy, respectively (Zeiss LSM700, Carl Zeiss, Inc., Thornwood, NY, USA).

To verify the tissue-specific expression of *ES2*, the promoter of *ES2* (2103 bp upstream of ATG) was amplified from WYG7 genomic DNA and inserted into the binary vector pCAMBIA1305 with the GUS reporter gene. The recombinant vector is then introduced into the callus of WYG7 to produce a transgenic plant. GUS assays were performed on different tissues of transgenic plants, including roots, stems, leaves, sheaths, and panicle.

### RNA extraction and quantitative real-time PCR (qRT-PCR) analysis

Total RNA samples were extracted from roots, stems, leaves, sheaths and panicles of wild-type WYG7 and *es2* plants using AxyPrep™ total RNA Miniprep Kit (Axygen) at the tillering stage. Then the total RNA was reverse transcribed into cDNA using the ReverTra Ace® quantitative PCR RT Master Mix gDNA remover Kit (Toyobo Co. Ltd.; http://www.toyobo.cn/). qRT-PCR analysis was conducted with CFX96 Touch™ Real-Time PCR (Bio-Rad). All target genes were tested for expression levels using the rice reference gene *Histone* (*LOC_Os06g04030*) as a standard. Three biological replicates were performed in this experiment and *t*-test was used for statistical analysis. The primers used for qRT-PCR are listed in Additional file 4: Table S4.

## Supplementary information

**Additional file 1: Table S1** Genetic analysis of the *es2* mutant in F_2_ population.

**Additional file 2: Table S2** Primers for fine mapping in this study.

**Additional file 3: Table S3** Primers for vector construction in this study.

**Additional file 4: Table S4** Primers for qRT-PCR in this study.

**Additional file 5: Figure S1**. Dark-induced senescence in the leaves of WYG7 and *es2.* (**a**, **b**) WYG7 leaves were incubated at 2-leaf stage in the dark for 0 and 5 d. (**c, d**) *es2* leaves were incubated at 2-leaf stage in the dark for 0 and 5 d.

**Additional file 6: Figure S2.** *ES2* affects rice yield components. (**a**) Phenotypes of *es2* and the wild-type (WYG7) at the mature stage (45 days after pollination). Scale bar = 10 cm. (**b**, **g**) Internode length of the main stem at the mature stage. Scale bar = 2 cm. (**c**, **h**) Panicle length of the main stem at the mature stage. Scale bar = 2 cm. (**d**, **k**) Grain width at the mature stage. Scale bar = 2 cm. (**e**) Plant height. (**f**) Tillering number. (**i**) Number of primary branch. (**j**) Number of secondary branch. (**l**) 1000-grain weight. Mean ± SD, *n* = 9. * significance at *P* < 5%, ** extremely significance at *P* < 1% (Student’s *t*-test).

**Additional file 7: Figure S3**. Photosynthetic parameters were restored in complementation and overexpression lines with *ES2*. (**a**, **b**, **c**) Photosynthetic parameters in flag leaf of WYG7, *es*2, COM-1, COM-2, OE-3 and OE-6 at the heading stage. Mean ± SD, *n* = 3. A, B indicate a significant difference at the 1% level (Student’s *t*-test).

**Additional file 8: Figure S4**. ES2 was localized to nucleus and plasma membrane in rice protoplasts and tobacco leaf epidermal cells. (**a**-**c**) Rice protoplast transformed with p35S::GFP as a control. Scale bar = 5 μm; (**d**-**f**) Rice protoplast transformed with p35S::ES2::GFP. Scale bar = 2 μm; (**g**-**i**) Tobacco (*N. benthamiana*) leaf epidermal cells transformed with p35S::GFP as a control. Scale bar = 20 μm; (**j**-**l**) Tobacco (*N. benthamiana*) leaf epidermal cells transformed with p35S::ES2::GFP. Scale bar = 20 μm.

**Additional file 9: Figure S5**. ES2 was localized to nucleus and plasma membrane in rice protoplasts transformed with p35S::YFP. (**a**-**d**) Rice protoplast transformed with p35S::YFP as a control. Scale bar = 5 μm; (**e**-**h**) Rice protoplast transformed with p35S::ES2::YFP. Scale bar = 5 μm.

**Additional file 10: Figure S6**. Tissue expression pattern of *ES2*. (**a**-**e**) GUS expression of transgenic rice with p*ES2*::GUS at the heading stage. (**a**) Root. (**b**) Stem. (**c**) Leaf. (**d**) Sheath. (**e**) Panicle. Scale bar = 2 cm. (**f**) Relative expression levels of *ES2* in various tissues revealed by qRT-PCR using *Histone* as the reference gene. Mean ± SD, *n* = 3.

**Additional file 11: Figure S7**. Expression levels of *OsIPK2* in leaves from the wild type (WYG7) and the *es2* mutants at seedling, tillering and heading stages. *Histone* gene was used as the reference. Mean ± SD, *n* = 3. * significance at *P* < 5%, ** extremely significance at *P* < 1% (Student’s *t*-test).

**Additional file 12: Figure S8**. Leaf phenotype and relative expression level of *OsIPK2* in seedlings of WYG7, *es2* and overexpression lines OE*es2*–1, OE*es2*–2, and OE*es2*–3. (**a**) WYG7, (**b**) *es2*, (**c**) OE*es2*–1, (**d**) OE*es2*–2, (**e**) OE*es2*–3. Scale bar = 2 cm. (**f**) The relative expression level of *OsIPK2* in WYG7, *es2* and overexpression lines OE*es2*–1, OE*es2*–2, and OE*es2*–3. Mean ± SD, *n* = 3. ** significance at *P* < 1% (Student’s *t*-test).

**Additional file 13: Figure S9**. Determination of phytic acid content in leaves from the wild-type (WYG7) and the *es2* mutants at seedling, tillering and heading stages. Mean ± SD, *n* = 3. * significance at *P* < 5%, ** significance at *P* < 1% (Student’s *t*-test).

## Data Availability

All relevant data are provided as figures or tables within the paper.

## Abbreviations

EMS: Ethyl methanesulfonate mutagenesis
Chl: Chlorophyll
Chl *a*: Chlorophyll *a*
Chl *b*: Chlorophyll *b*
Car: Carotenoid
TEM: Transmission electron microscopy
NBT: Nitro blue tetrazolium
DAB: 3,3′-diaminobenzidine
qRT-PCR: Quantitative real-time PCR
*P*_n_: Net photosynthetic rate
*T*_r_: Transpiration rate
*G*_s_: Stomatal conductance
ROS: Reactive oxygen species
O_2_^−^: Superoxide anions
H_2_O_2_: Hydrogen peroxide
MDA: Malondialdehyde
CAT: Catalase
SOD: Superoxide dismutase
POD: Peroxidase
APX: Ascorbate peroxidase
TUNEL: Terminal deoxynucleotidyl transferase-mediated dUTP nick-end labeling
PI: Propidium iodide
CDS: Coding sequence

## References

[1] Koyama T (2014). The roles of ethylene and transcription factors in the regulation of onset of leaf senescence. Front Plant Sci.

[2] Munne-Bosch S (2008). Do perennials really senesce?. Trends Plant Sci.

[3] Leng Y, Yang Y, Ren D, Huang L, Dai L, Wang Y (2017). A rice *PECTATELYASE-LIKE* gene is required for plant growth and leaf senescence. Plant Physiol.

[4] Mitchell PL, Sheehy JE (2006). Super charging rice photosynthesis to increase yield. New Phytol.

[5] Yang Y, Xu J, Huang L, Leng Y, Dai L, Guo L (2015). PGL, encoding chlorophyllide a oxygenase 1, impacts leaf senescence and indirectly affects grain yield and quality in rice. J Exp Bot.

[6] Mao C, Lu S, Lv B, Zhang B, Shen J, He J (2017). A rice NAC transcription factor promotes leaf senescence via ABA biosynthesis. Plant Physiol.

[7] Wang XJ, Xu QG, Yang ZJ (2005). Advances of research on rice leaf senescence physiology (in Chinese). Chinese Agric Sci Bull.

[8] Liang JS, Cao XZ (1993). Studies of the relationship between several physiological characteristics of leaf and bleeding rate of roots in hybrid rice (*O. sativa L.*) (in Chinese). Jiangsu Agric Sci.

[9] Guo Y, Gan SS (2014). Translational researches on leaf senescence for enhancing plant productivity and quality. J Exp Bot.

[10] Lim PO, Kim HJ, Nam HG (2007). Leaf senescence. Annu Rev Plant Biol.

[11] Yamada Y, Furusawa S, Nagasaka S, Shimomura K, Yamaguchi S, Umehara M (2014). Strigolactone signaling regulates rice leaf senescence in response to a phosphate deficiency. Planta..

[12] Wu L, Ren D, Hu S, Li G, Dong G, Jiang L (2016). Down-regulation of a nicotinate phosphoribosyl transferase gene, *OsNaPRT1*, leads to withered leaf tips. Plant Physiol.

[13] Yang X, Gong P, Li K, Huang F, Cheng F, Pan G (2016). A single cytosine deletion in the *OsPLS1* gene encoding vacuolar-type H^+^-ATPase subunit A1 leads to premature leaf senescence and seed dormancy in rice. J Exp Bot.

[14] Yang X, Nian J, Xie Q, Feng J, Zhang F, Jing H (2016). Rice ferredoxin-dependent glutamate synthase regulates nitrogen-carbon metabolomes and is genetically differentiated between *japonica* and *indica* subspecies. Mol Plant.

[15] Zhao Y, Chan Z, Gao J, Xing L, Cao M, Yu C (2016). ABA receptor PYL9 promotes drought resistance and leaf senescence. Proc Natl Acad Sci U S A.

[16] Deng L, Qin P, Liu Z, Wang G, Chen W, Tong J (2016). Characterization and fine-mapping of a novel premature leaf senescence mutant *yellow leaf and dwarf 1* in rice. Plant Physiol Biochem.

[17] Hong Y, Zhang Y, Sinumporn S, Yu N, Zhan X, Shen X (2018). Premature leaf senescence 3, encoding a methyltransferase, is required for melatonin biosynthesis in rice. Plant J.

[18] Ke S, Liu S, Luan X, Xie XM, Hsieh TF, Zhang XQ (2019). Mutation in a putative glycosyltransferase-like gene causes programmed cell death and early leaf senescence in rice. Rice.

[19] Jiao BB, Wang JJ, Zhu XD, Zeng LJ, Li Q, He ZH (2012). A novel protein RLS1 with NB-ARM domains is involved in chloroplast degradation during leaf senescence in rice. Mol Plant.

[20] Lee RH, Lin MC, Chen SC (2004). A novel alkaline α-galactosidase gene is involved in rice leaf senescence. Plant Mol Biol.

[21] Kusaba M, Ito H, Morita R, Iida S, Sato Y, Fujimoto M (2007). Rice NON-YELLOW Coloring1 is involved in light-harvesting complex II and grana degradation during leaf senescence. Plant Cell.

[22] Morita R, Sato Y, Masuda Y, Nishimura M, Kusaba M (2009). Defect in *non-yellow coloring 3*, an α/β hydrolase-fold family protein, causes a stay-green phenotype during leaf senescence in rice. Plant J.

[23] Sato Y, Morita R, Katsuma S, Nishimura M, Tanaka A, Kusaba M (2009). Two short-chain dehydrogenase/reductases, NON-YELLOW COLORING 1 and NYC1-LIKE, are required for chlorophyll b and light-harvesting complex II degradation during senescence in rice. Plant J.

[24] Rong H, Tang Y, Zhang H, Wu P, Chen Y, Li M (2013). The stay-green Rice like (SGRL) gene regulates chlorophyll degradation in rice. J Plant Physiol.

[25] Liang C, Wang Y, Zhu Y, Tang J, Hu B, Liu L (2014). OsNAP connects abscisic acid and leaf senescence by fine-tuning abscisic acid biosynthesis and directly targeting senescence-associated genes in rice. Proc. Natl Acad. Sci U S A.

[26] Chen H, Cheng Z, Ma X, Wu H, Liu Y, Zhou K (2013). A knockdown mutation of *YELLOW-GREEN LEAF2* blocks chlorophyll biosynthesis in rice. Plant Cell Rep.

[27] Havlová M, Dobrev PI, Motyka V, Storchová H, Libus J, Dobrá J (2008). The role of cytokinins in responses to water deficit in tobacco plantsover-expressing trans-zeatin O-glucosyltransferase gene under 35S or SAG12 promoters. Plant Cell Environ.

[28] Kai H, Wei W, Su-Sheng G (2012). SAUR36, a small auxin up RNA gene, is involved in the promotion of leaf senescence in Arabidopsis. Plant Physiol.

[29] Ansari MI, Lee RH, Chen SCG (2004). A novel senescence-associated gene encoding gamma-aminobutyric acid (GABA): pyruvate transaminase is upregulated during rice leaf senescence. Physiol Plant.

[30] Sun L, Wang Y, Liu LL, Wang C, Gan T, Zhang Z (2017). Isolation and characterization of a *spotted leaf 32* mutant with early leaf senescence and enhanced defense response in rice. Sci Rep.

[31] Zhou Y, Liu L, Huang W, Yuan M, Zhou F, Li X (2014). Overexpression of *OsSWEET5* in rice causes growth retardation and precocious senescence. PLoS One.

[32] Wang Z, Wang Y, Hong X, Hu D, Liu C, Yang J (2015). Functional inactivation of UDP-N-acetylglucosamine pyrophosphorylase 1 (UAP1) induces early leaf senescence and defence responses in rice. J Exp Bot.

[33] Rao Y, Yang Y, Xu J, Li X, Leng Y, Dai L (2015). *EARLY SENESCENCE 1* encodes a SCAR-like protein2 that affects water loss in rice. Plant Physiol.

[34] Lin HC, Karki S, Coe RA, Bagha S, Khoshravesh R, Balahadia CP (2016). Targeted knockdown of GDCH in rice leads to a photorespiratory deficient phenotype useful as a building block for C 4 rice. Plant Cell Physiol.

[35] He Y, Zhang Z, Li L, Tang S, Wu J (2018). Genetic and physio-biochemical characterization of a novel premature senescence leaf mutant in rice (*Oryza sativa L.*). Int J Mol Sci.

[36] Bennett M, Onnebo SM, Azevedo C, Saiardi A (2006). Inositol pyrophosphates: metabolism and signaling. Cell Mol Life Sci.

[37] Michell RH (2008). Inositol derivatives: evolution and functions. Nat Rev Mol Cell Biol.

[38] Endo-Streeter S, Tsui MK, Odom AR, Block J, York JD (2012). Structural studies and protein engineering of inositol phosphate multikinase. J Biol Chem.

[39] Shears SB (2004). How versatile are inositol phosphate kinases?. Biochem J.

[40] Xia HJ, Brearley C, Elge S, Kaplan B, Fromm H, Mueller-Roeber B (2003). Arabidopsis inositol polyphosphate 6−/3-kinase is a nuclear protein that complements a yeast mutant lacking a functional ArgR-Mcm1 transcription complex. Plant Cell.

[41] Stevenson-Paulik J, Odom AR, York JD (2002). Molecular and biochemical characterization of two plant inositol polyphosphate 6−/3−/5-kinases. J Biol Chem.

[42] Bosch D, Saiardi A (2012). Arginine transcriptional response does not require inositol phosphate synthesis. J Biol Chem.

[43] Li J, Zhang B, Ma T, Wang H, Zhang B, Yu Q (2017). Role of the inositol polyphosphate multikinase Ipk2 in regulation of hyphal development, calcium signaling and secretion in *Candida albicans*. Mycopathologia..

[44] Saiardi A, Sciambi C, McCaffery JM, Wendland B, Snyder SH (2002). Inositol pyrophosphates regulate endocytic trafficking. Proc Natl Acad Sci U S A.

[45] Saiardi A, Resnick AC, Snowman AM, Wendland B, Snyder SH (2005). Inositol pyrophosphates regulate cell death and telomere length through phosphoinositide 3-kinase-related protein kinases. Proc Natl Acad Sci U S A.

[46] Banfic H, Bedalov A, York JD, Visnjic D (2013). Inositol pyrophosphates modulate S phase progression after pheromone-induced arrest in *Saccharomyces cerevisiae*. J Biol Chem.

[47] Zhang ZB, Yang G, Arana F, Chen Z, Li Y, Xia HJ (2007). *Arabidopsis* inositol polyphosphate 6−/3-kinase (AtIpk2β) is involved in axillary shoot branching via auxin signaling. Plant Physiol.

[48] Xu J, Brearley CA, Lin WH, Wang Y, Ye R, Mueller-Roeber B (2005). A role of *Arabidopsis* inositol polyphosphate multikinase, AtIPK2α, in pollen germination and root growth. Plant Physiol.

[49] Baena-Gonzalez E, Rolland F, Thevelein JM, Sheen J (2007). A central integrator of transcription networks in plant stress and energy signalling. Nature..

[50] Cho YH, Hong JW, Kim EC, Yoo SD (2012). Regulatory functions of SnRK1 in stress-responsive gene expression and in plant growth and development. Plant Physiol.

[51] Tsai AY, Gazzarrini S (2012). AKIN10 and FUSCA3 interact to control lateral organ development and phase transitions in arabidopsis. Plant J.

[52] Tsai AY, Gazzarrini S (2012). Overlapping and distinct roles of AKIN10 and FUSCA3 in ABA and sugar signaling during seed germination. Plant Signal Behav.

[53] Yang Q, Sang S, Chen Y, Wei Z, Wang P (2017). The role of *Arabidopsis* inositol polyphosphate multikinase AtIPK2β in glucose suppression of seed germination and seedling development. Plant Cell Physiol.

[54] Suzuki M, Tanaka K, Kuwano M, Yosgida K (2007). T. Expression pattern of inositol phosphate-related enzymes in rice (*Oryza sativa L*.): implications for the phytic acid biosynthetic pathway. Gene..

[55] Xu R, Sen N, Paul BD, Snowman AM, Pao F, Vandiver MS (2013). Inositol polyphosphate multikinase is a coactivator of p53-mediated transcription and cell death. Sci Signal.

[56] Lee RH, Wang CH, Huang LT, Chen SCG (2001). Leaf senescence in rice plants: cloning and characterization of senescence up-regulated genes. J Exp Bot.

[57] Xie Z, Zhang Z, Zou X, Huang J, Ruas P, Thompson D (2005). Annotations and functional analyses of the Rice WRKY gene superfamily reveal positive and negative regulators of abscisic acid signaling in Aleurone cells. Plant Physiol.

[58] Shen J, Lv B, Luo L, He J, Mao C, Xi D (2017). The NAC-type transcription factor OsNAC2 regulates ABA-dependent genes and abiotic stress tolerance in rice. Sci Rep.

[59] Saika H, Ohtsu K, Hamanaka S, Nakazono M, Tsutsumi N, Hirai A (2002). AOX1c, a novel rice gene for alternative oxidase; comparison with rice AOX1a and AOX1b. Genes Genet Syst.

[60] Agrawal GK, Jwa NS, Iwahashi H, Rakwal P (2003). Importance of ascorbate peroxidases OsAPX1 and OsAPX2 in the rice pathogen response pathways and growth and reproduction revealed by their transcriptional profiling. Gene.

[61] Ye N, Zhu G, Liu Y, Li Y, Zhang J (2011). ABA controls H_2_O_2_ accumulation through the induction of OsCATB in Rice leaves under water stress. Plant Cell Physiol.

[62] Goslings D, Meskauskiene R, Kim C, Lee KP, Nater M, Apel K (2004). Concurrent interactions of heme and FLU with Glu tRNA reductase (*HEMA1*), the target of metabolic feedback inhibition of tetrapyrrole biosynthesis, in dark- and light-grown Arabidopsis plants. Plant J.

[63] Zhang H, Li J, Yoo J, Yoo S, Cho S, Koh H (2006). Rice Chlorina-1 and Chlorina-9 encode ChlD and ChlI subunits of mg-chelatase, a key enzyme for chlorophyll synthesis and chloroplast development. Plant Mol Biol.

[64] Wang P, Gao J, Wan C, Zhang F, Xu Z, Huang X (2010). Divinyl chlorophyll (ide) a can be converted to monovinyl chlorophyll (ide) a by a divinyl reductase in rice. Plant Physiol.

[65] Wang P, Deng X (2013). One divinyl reductase reduces the 8-vinyl groups in various intermediates of chlorophyll biosynthesis in a given higher plant species, but the isozyme differs between species. Plant Physiol.

[66] Ohmiya A, Hirashima M, Yagi M, Tanase K, Yamamizo C (2014). Identification of genes associated with chlorophyll accumulation in flower petals. PLoS One.

[67] Lee S, Kim JH, Yoo ES, Lee CH, Hirochika H, An G (2005). Differential regulation of chlorophyll a oxygenase genes in rice. Plant Mol Biol.

[68] Tang Y, Li M, Chen Y, Wu P, Wu G, Jiang H (2011). Knockdown of *OsPAO* and *OsRCCR1* cause different plant death phenotypes in rice. J Plant Physiol.

[69] Caffarri S, Croce R, Cattivelli L, Bassi R (2004). A look within LHCII: differential analysis of the Lhcb1-3 complexes building the major trimeric antenna complex of higher-plant photosynthesis. Biochemistry..

[70] Mei J, Li F, Liu X, Hu G, Fu Y, Liu W (2017). Newly identified CSP41b gene localized in chloroplasts affects leaf color in rice. Plant Sci.

[71] Sugimoto H, Kusumi K, Noguchi K, Yano M, Yoshimura A, Iba K (2007). The rice nuclear gene, *VIRESCENT 2*, is essential for chloroplast development and encodes a novel type of guanylate kinase targeted to plastids and mitochondria. Plant J.

[72] Lin Y, Tan L, Zhao L, Sun X, Sun C (2016). RLS3, a protein with AAA+ domain localized in chloroplast, sustains leaf longevity in rice. J Integr Plant Biol.

[73] Wang B, Zhang Y, Bi Z, Liu Q, Xu T, Yu N (2019). Impaired function of the calcium-dependent protein kinase, *OsCPK12*, leads to early senescence in rice (*Oryza sativa* L.). Front Plant Sci.

[74] Yang L, Tang RJ, Zhu JQ, Liu H, Mueller-Roeber B, Xia HJ (2008). Enhancement of stress tolerance in transgenic tobacco plants constitutively expressing AtIpk2β, an inositol polyphosphate 6−/3-kinase from Arabidopsis thaliana. Plant Mol Biol.

[75] Zhan HD, Zhong YJ, Yang ZN, Xia HJ (2015). Enzyme activities of Arabidopsis inositol polyphosphate kinases AtIPK2α and AtIPK2β are involved in pollen development, pollen tube guidance and embryogenesis. Plant J.

[76] Chen Y, Yang Q, Sang S, Wei Z, Wang P (2017). Rice inositol polyphosphate multikinase (OsIPK2) directly interacts with OsIAA11 to regulate lateral root formation. Plant Cell Physiol.

[77] Chen Y, Wei Z, Yang Q, Sang S, Wang P (2017). Rice inositol polyphosphate multikinase gene (*OsIPK2*), a putative new player of gibberellic acid signaling, involves in modulation of shoot elongation and fertility. Plant Cell Tiss Org.

[78] Han M, Kim C, Lee J, Lee S, Jeon J (2014). *OsWRKY42* represses *OsMT1d* and induces reactive oxygen species and leaf senescence in rice. Mol Cell.

[79] Brodersen P, Petersen M, Pike HM, Olszak B, Skov B, Odum N (2002). Knockout of *Arabidopsis ACCELERATED-CELL-DEATH11* encoding a sphingosine transfer protein causes activation of programmed cell death and defense. Genes Dev.

[80] Cui MH, Ok SH, Yoo KS, Jung KW, Yoo SD, Shin JS (2013). An *Arabidopsis* cell growth defect factor-related protein, CRS, promotes plant senescence by increasing the production of hydrogen peroxide. Plant Cell Physiol.

[81] Zhou Q, Yu Q, Wang Z, Pan Y, Lv W, Zhu L (2013). Knockdown of *GDCH* gene reveals reactive oxygen species-induced leaf senescence in rice. Plant Cell Environ.

[82] Wang M, Zhang T, Peng H, Luo S, Tan J, Jiang K (2018). Rice *Premature Leaf Senescence 2*, encoding a glycosyltransferase (GT), is involved in leaf senescence. Front Plant Sci.

[83] Scandalios JG (2002). The rise of ROS. Trends Biochem Sci.

[84] Mittler R, Vanderauwera S, Gollery M, Van Breuseqem F (2004). Reactive oxygen gene network of plants. Trends Plant Sci.

[85] Ribeiro CW, Korbes AP, Garighan JA, Jardim-Messeder D, FEL C, RHV S (2017). Rice peroxisomal ascorbate peroxidase knockdown affects ROS signaling and triggers early leaf senescence. Plant Sci.

[86] Miller GN, Ciftci-Yilmaz S, Mittler R (2010). Reactive oxygen species homeostasis and signalling during drought and salinity stresses. Plant Cell Environ.

[87] Pandey V, Shukla A (2015). Acclimation and tolerance strategies of rice under drought stress. Rice Sci.

[88] Saleethong P, Roytrakul S, Kong-Ngern K, Theerakulpisut P (2016). Differential proteins expressed in rice leaves and grains in response to salinity and exogenous spermidine treatments. Rice Sci.

[89] Su Y, Hu S, Zhang B, Ye W, Niu Y, Guo L (2017). Characterization and fine mapping of a new early leaf senescence mutant *es3(t)* in rice. Plant Growth Regul.

[90] Bi Z, Zhang Y, Wu W, Zhan X, Yu N, Xu T (2017). *ES7*, encoding a ferredoxin-dependent glutamate synthase, functions in nitrogen metabolism and impacts leaf senescence in rice. Plant Sci.

[91] Zhao C, Liu C, Zhang Y, Cui Y, Hu H, Noushin J (2019). A 3-bp deletion of *WLS5*, gene leads to weak growth and early leaf senescence in rice. Rice..

[92] Ramel F, Sulmon C, Bogard M, Couée I, Gouesbet G (2009). Differential patterns of reactive oxygen species and antioxidative mechanisms during atrazine injury and sucrose-induced tolerance in*Arabidopsis* thalianaplantlets. BMC Plant Biol.

[93] Kong X, Li D (2011). Hydrogen peroxide is not involved in HrpN from Erwinia amylovora-induced hypersensitive cell death in maize leaves. Plant Cell Rep.

[94] Huang YJ, Lu KS (2001). TUNEL staining and electron microscopy studies of apoptotic changes in the Guinea pig vallate taste cells after unilateral glossopharyngeal denervation. Anat Embryol (Berl).

[95] Ren D, Yu H, Rao Y, Xu Q, Zhou T, Hu J (2017). ‘Two-floret spikelet’ as a novel resource has the potential to increase rice yield. Plant Biotechnol J.

[96] Ruan B, Hua Z, Zhao J, Zhang B, Ren D, Liu C (2019). OsACL-A2 negatively regulates cell death and disease resistance in rice. Plant Biotechnol J.

