# Supplementary material for: Rice EARLY SENESCENCE 2, encoding an inositol polyphosphate kinase, is involved in leaf senescence
Source: BMC Plant Biol. 2020 Aug 26;20:393. doi: 10.1186/s12870-020-02610-1 (PMC7449006; doi:10.1186/s12870-020-02610-1)
Supplement: Supplementary file 1 — Additional file 1: Table S1 Genetic analysis of the es2 mutant in F2 population. [file 12870_2020_2610_MOESM1_ESM.doc]

**Table S1 Genetic analysis of the *es2* mutant in F_2_ population.**

| Cross | F_1_ | F_2_ | | χ^2^ (3:1) | P-Value |
| --- | --- | --- | --- | --- | --- |
|  |  | Wild-type | Mutant |  |  |
| *es2*/WYG7 | Normal | 604 | 180 | 1.7361 | 0.1876 |
| *es2*/93-11 | Normal | 1537 | 521 | 0.1095 | 0.7407 |
