# Supplementary material for: Rice EARLY SENESCENCE 2, encoding an inositol polyphosphate kinase, is involved in leaf senescence
Source: BMC Plant Biol. 2020 Aug 26;20:393. doi: 10.1186/s12870-020-02610-1 (PMC7449006; doi:10.1186/s12870-020-02610-1)
Supplement: Supplementary file 2 — Additional file 2: Table S2 Primers for fine mapping in this study. [file 12870_2020_2610_MOESM2_ESM.doc]

**Table S2 Primers for fine mapping in this study.**

| Marker | Forward primer sequence (5’-3’) | Reverse primer sequence (5’-3’) |
| --- | --- | --- |
| B2-9 | TGACTATGTAAGGTTGCTGCTG | ACAGCTCCACAGCAAAGAACC |
| B2-10 | GTTGATAGATTGCATTGCCAA | CAAAGCTAGAAGTGTTAACCTGTAA |
| B2-11 | CATGGTCATCGCAACGCATCATTCG | GCTCCAGCAGCCGTCCCTTGAGCTA |
| ID2-1 | ACAGCCATCTAGCCTGCCTA | AGAAAATATCATGGGAAACCCA |
| ID2-2 | CGGAGGCAATCTCTCACAAT | TCTGCCGTAGCTTTCTTTGG |
| ID2-3 | TTGTGCCTTGTGAGTTGTTTAC | TCCAACTGACAACACAAACACT |
| ID2-4 | GCTTTGTTTCTTGTGGGATACAGG | CTTTCCGGTGTCAATCCAAGC |
| ID2-5 | GGCCTCTCTCACCACACA | ACTGTGAGAGGAGGAAGCTAAG |
| ID2-6 | AGCTCAGCTCGTTGCTCTTC | GCACAACACCACAAAACACC |
| ID2-7 | CCATGTCCACGCTATCCTTT | GAAAAATGGATGCTTGTGGG |
