# Supplementary material for: Rice EARLY SENESCENCE 2, encoding an inositol polyphosphate kinase, is involved in leaf senescence
Source: BMC Plant Biol. 2020 Aug 26;20:393. doi: 10.1186/s12870-020-02610-1 (PMC7449006; doi:10.1186/s12870-020-02610-1)
Supplement: Supplementary file 3 — Additional file 3: Table S3 Primers for vector construction in this study. [file 12870_2020_2610_MOESM3_ESM.doc]

**Table S3 Primers for vector construction in this study.**

| Primer | Sequence（5’→3’） | Site |
| --- | --- | --- |
| ES2-COM-F | acgaattcgagctcggtaccttagtcccgattatttcacccg | KpnⅠ |
| ES2-COM-R | tcgactctagaggatcctagatccggtgccacatctata | BamHⅠ |
| ES2-OE-F | ttcgagctcGGTACCATGGCCTCCGACCTGCGCCCGC | KpnⅠ |
| ES2-OE-R | gcaggtcgacTCTAGATCAAGAATGATCTGAAGACGCC | XbaⅠ |
| ES2-GUS-F | GCAGGCATGCAAGCTTttagtcccgattatttcacccg | HindⅢ |
| ES2-GUS-R | CTCAGATCTACCATGGggcggcggcggcggcggcggcg | NcoⅠ |
| ES2-GFP-F | TTACAATTACAGTCGAATGGCCTCCGACCTGCGCCCGC | SalⅠ |
| ES2-GFP-R | TGGATCCTCTAGAGTCAGAATGATCTGAAGACGCCTTC |  |
| ES2-YFP-F | AAAAGATCTATGGCCTCCGACCTGCGCCCGC | BglⅡ |
| ES2-YFP-R | AAAAGATCTTCAAGAATGATCTGAAGACGCC |  |
