# Supplementary material for: Rice EARLY SENESCENCE 2, encoding an inositol polyphosphate kinase, is involved in leaf senescence
Source: BMC Plant Biol. 2020 Aug 26;20:393. doi: 10.1186/s12870-020-02610-1 (PMC7449006; doi:10.1186/s12870-020-02610-1)
Supplement: Supplementary file 4 — Additional file 4: Table S4 Primers for qRT-PCR in this study. [file 12870_2020_2610_MOESM4_ESM.doc]

**Table S4 Primers for qRT-PCR in this study.**

| Gene | Forward primer sequence (5’-3’) | Reverse primer sequence (5’-3’) |
| --- | --- | --- |
| *Osh36* | GCACGGAGGCGAACGA | TTGAGCGGTAGCACCCATT |
| *OsI57* | ACCCTAAAGTAAATGAAGTC | CCTGCTCTTGTCTTGTTA |
| *OsI85* | GAGCAACGGCGTGGAGA | GCGGCGGTAGAGGAGATG |
| *OsWRKY23* | TCCAGTTCCTCTCCCAGTTCTAA | CACATTGTTCTCCTTTTCTTCCC |
| *OsWRKY72* | CACCACAAATCACATCTACTCCG | GCTGAAGGGAAGAGAGGTGAG |
| *OsNAC2* | AAAAACAACCGCATTGGCAG | AGTCCTCATCTCCTCTGTCTAATCC |
| *SGR* | AGGGGTGGTACAACAAGCTG | GCTCCTTGCGGAAGATGTAG |
| *AOX1a* | CTTCGCATCGGACATCCATTA | TCCTCGGCAGTAGACAAACATC |
| *AOX1b* | CCTGCTCAGTTCATCACCATCA | GCATAAAACGGAGTGACAATAGC |
| *APX1* | AGGTGCCACAAGGAAAGATCTGGT | TCAGCAGGGCTTTGTCACTAGGAA |
| *APX2* | TGGGAAGATGCCACAAGGAGAGAT | TCCGCAGCATATTTCTCCACCAGT |
| *SODB* | TCCGCCGTATAAACTTGATGCCCT | TGGGTTGCCGTTGTTGTATGCTTC |
| *SODA1* | ATCTGGATGGGTGTGGCTAGCTTT | AGTACGCATGCTCCCAGACATCAA |
| *CATA* | CAACCGCAACGTCGACAACTTCTT | TTCACCGGCAGCATCAGGTAGTTT |
| *CATB* | GCTTGCTTTCTGCCCAGCGATAAT | AAATAGTTTGGGCCAAGACGGTGC |
| *HEMA* | ATGGAGGCCCAAACAATCATC | GCGTAGGACCTCAGCTTCTTGA |
| *GSA* | GCTCTCCGTGACTTGACGAAAC | CCGAAGTATTCTTGAGCCCCA |
| *HEME1* | GATCCCTTGAGAACAGCAGCTG | CATTGTTAACCTCTTCCCGCAA |
| *CHLD* | CGCATGCAGAATGCGAAAG | CCTCAGCAAAATCTCCACGAAA |
| *DVR* | CGAGCCCAGGTTCATCAAGGTGC | CCTCCCGATCTTGCCGAACTCC |
| *CHLH* | AACTGGATGAGCCAGAAGAGA | AAATGCAAAAGACTTGCGACT |
| *PORA* | ATGGCTCTCCAAGTTCAG | TGGCTCACGCTAAGGAAC |
| *PORB* | CCGCAAGGAGGGAGCGGTG | CCCTCTTGGTGCTAAGGCCG |
| *CAO1* | GACACCTTCATCTGGGCTTCAA | CGAGAGACATCCGGTAGAGC |
| *NYC1* | CATGCAACACCAACAAAAGG | GACCATTCCAGGAGAAGCAG |
| *NYC3* | tctatctaggtgccaaaggc | attctggcacctgctgtttc |
| *NOL* | CCACGAAAGGTATAGGATATG | TCAAGTCAGTCACCGCAGAT |
| *Rccr1* | CGCATTTCCTCATGGAATTT | CTTCTCACGCTGTTTGTCCA |
| *PCCR* | GGATCGACGATTGATTTCATG | GTCGAGGCGTTCAGAAAGAT |
| *RbcL* | CTTGGCAGCATTCCGAGTAA | ACAACGGGCTCGATGTGATA |
| *rbcS* | TCCGCTGAGTTTTGGCTATTT | GGACTTGAGCCCTGGAAGG |
| *psaA* | GCGAGCAAATAAAACACCTTTC | GTACCAGCTTAACGTGGGGAG |
| *psbA* | CCCTCATTAGCAGATTCGTTTT | ATGATTGTATTCCAGGCAGAGC |
| *CAB1R* | AGATGGGTTTAGTGCGACGAG | TTTGGGATCGAGGGAGTATTT |
| *CAB2R* | TGTTCTCCATGTTCGGCTTCT | GCTACGGTCCCCACTTCACT |
| *LchP2* | GAAGAAGATCAAGAACGGCC | TTGCCGGGGACGAAGTTGGT |
| *V2* | AGCAGATCCGTGATTACATGGCGA | TGCCTCTTCACTCTCTGCAACCAA |
| *RpoC1* | TCCGTCGGAACAACAATCTTG | TCCACGGCTTCTTGTACCAAT |
| *RpoC2* | ATGCATCGCAGGTACACCAA | CCCTCGCGTAAATTGCTTTG |
| *Rps15* | AGATACGGAGACTTGCTTCA | GCTCCCTAATATCCAACTGACT |
| *Lhcb1* | CCATGTTCTCCATGTTCGGCTTCT | TAGGCCCAGGCGTTGTTGTTGA |
| *Lhcb4* | TACCTGCAGTTCGAGCTGGAC | AGGCCGAACACCTCGGTGTA |
| *ES2* | GTGTAAGGGTGAAGCTGGTG | AATGTCGCCGATGAACTTGAT |
| *Histone* | GGTCAACTTGTTGATTCCCCTCT | AACCGCAAAATCCAAAGAACG |
