# Supplementary figures and images for: Rice EARLY SENESCENCE 2, encoding an inositol polyphosphate kinase, is involved in leaf senescence
Source: BMC Plant Biol. 2020 Aug 26;20:393. doi: 10.1186/s12870-020-02610-1 (PMC7449006; doi:10.1186/s12870-020-02610-1)

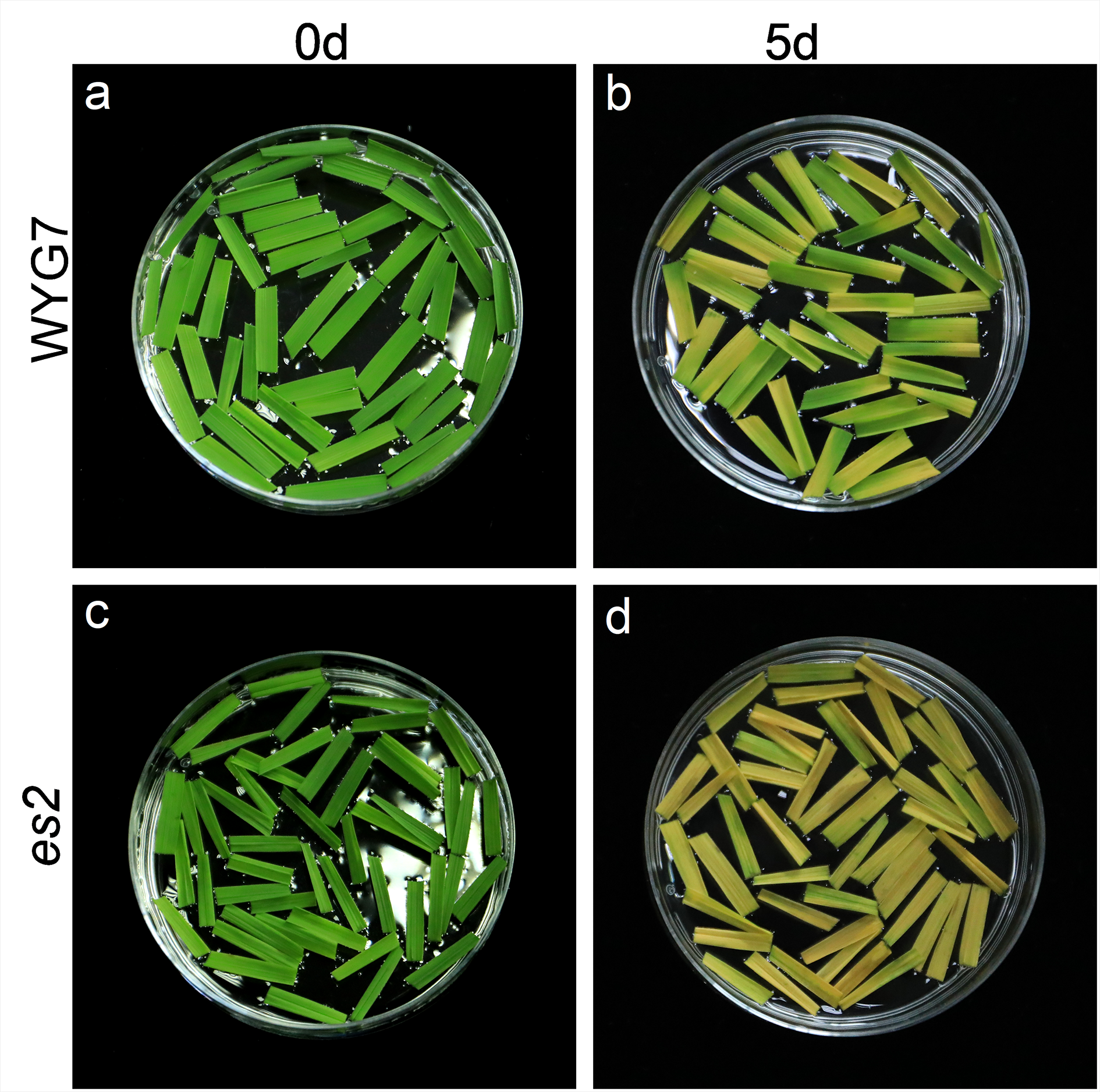

Supplement: Supplementary file 5 — Additional file 5: Figure S1. Dark-induced senescence in the leaves of WYG7 and es2. (a, b) WYG7 leaves were incubated at 2-leaf stage in the dark for 0 and 5 d. (c, d) es2 leaves were incubated at 2-leaf stage in the dark for 0 and 5 d. [file 12870_2020_2610_MOESM5_ESM.tif]

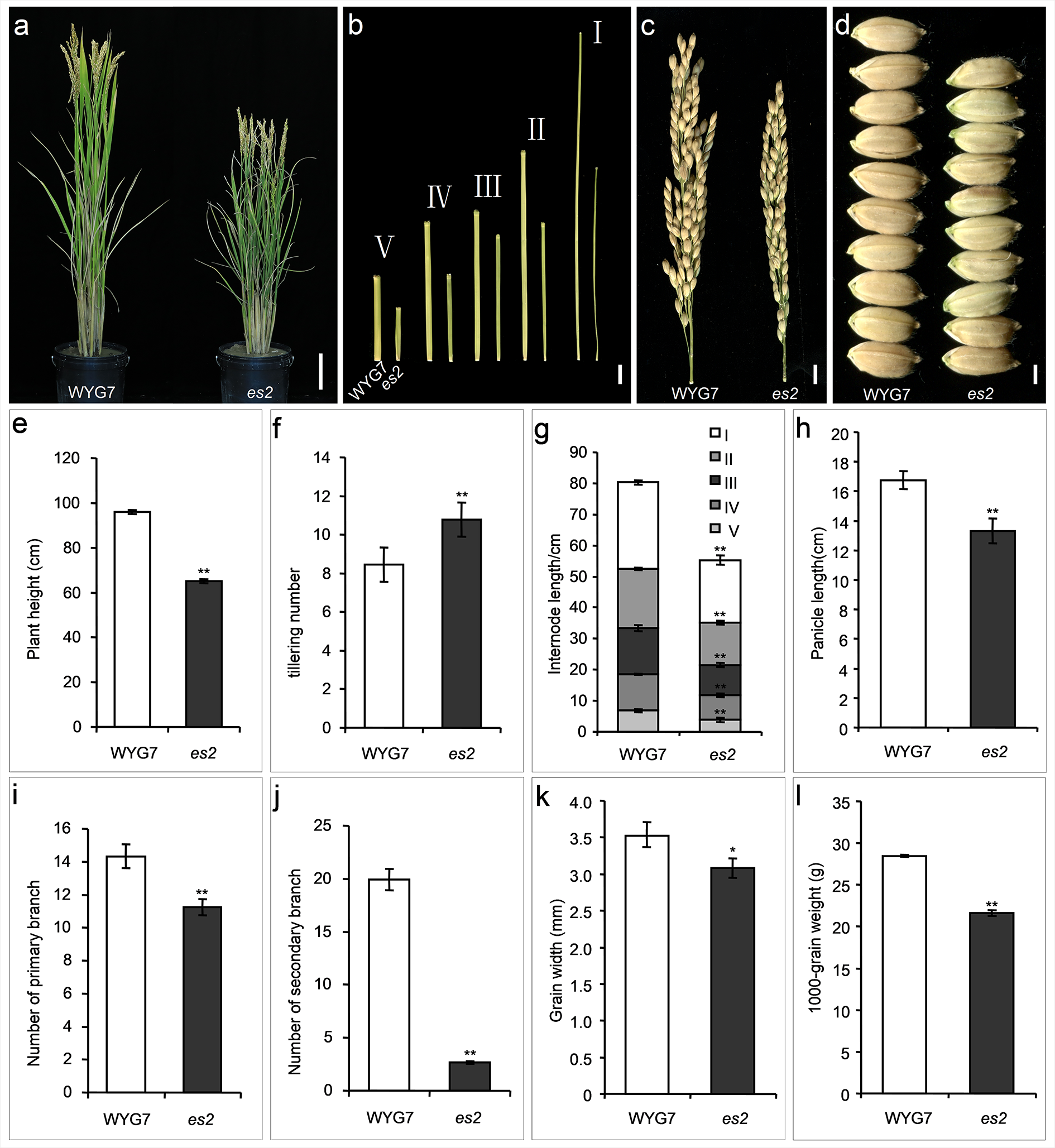

Supplement: Supplementary file 6 — Additional file 6: Figure S2. ES2 affects rice yield components. (a) Phenotypes of es2 and the wild-type (WYG7) at the mature stage (45 days after pollination). Scale bar = 10 cm. (b, g) Internode length of the main stem at the mature stage. Scale bar = 2 cm. (c, h) Panicle length of the main stem at the mature stage. Scale bar = 2 cm. (d, k) Grain width at the mature stage. Scale bar = 2 cm. (e) Plant height. (f) Tillering number. (i) Number of primary branch. (j) Number of secondary branch. (l) 1000-grain weight. Mean ± SD, n = 9. * significance at P < 5%, ** extremely significance at P < 1% (Student’s t-test). [file 12870_2020_2610_MOESM6_ESM.tif]

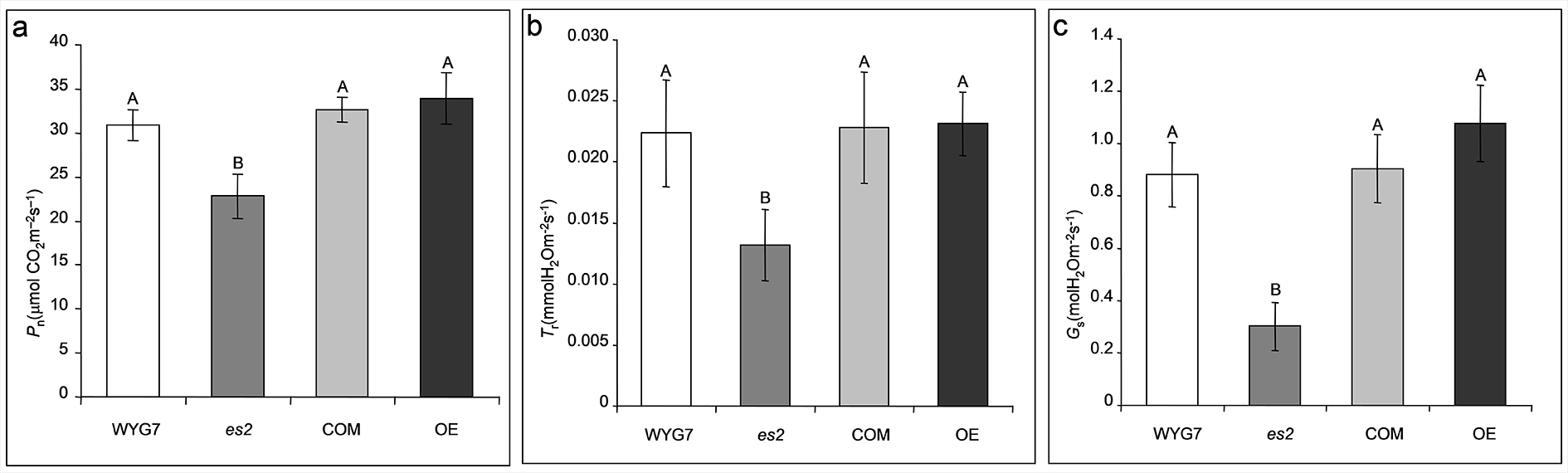

Supplement: Supplementary file 7 — Additional file 7: Figure S3. Photosynthetic parameters were restored in complementation and overexpression lines with ES2. (a, b, c) Photosynthetic parameters in flag leaf of WYG7, es2, COM-1, COM-2, OE-3 and OE-6 at the heading stage. Mean ± SD, n = 3. A, B indicate a significant difference at the 1% level (Student’s t-test). [file 12870_2020_2610_MOESM7_ESM.tif]

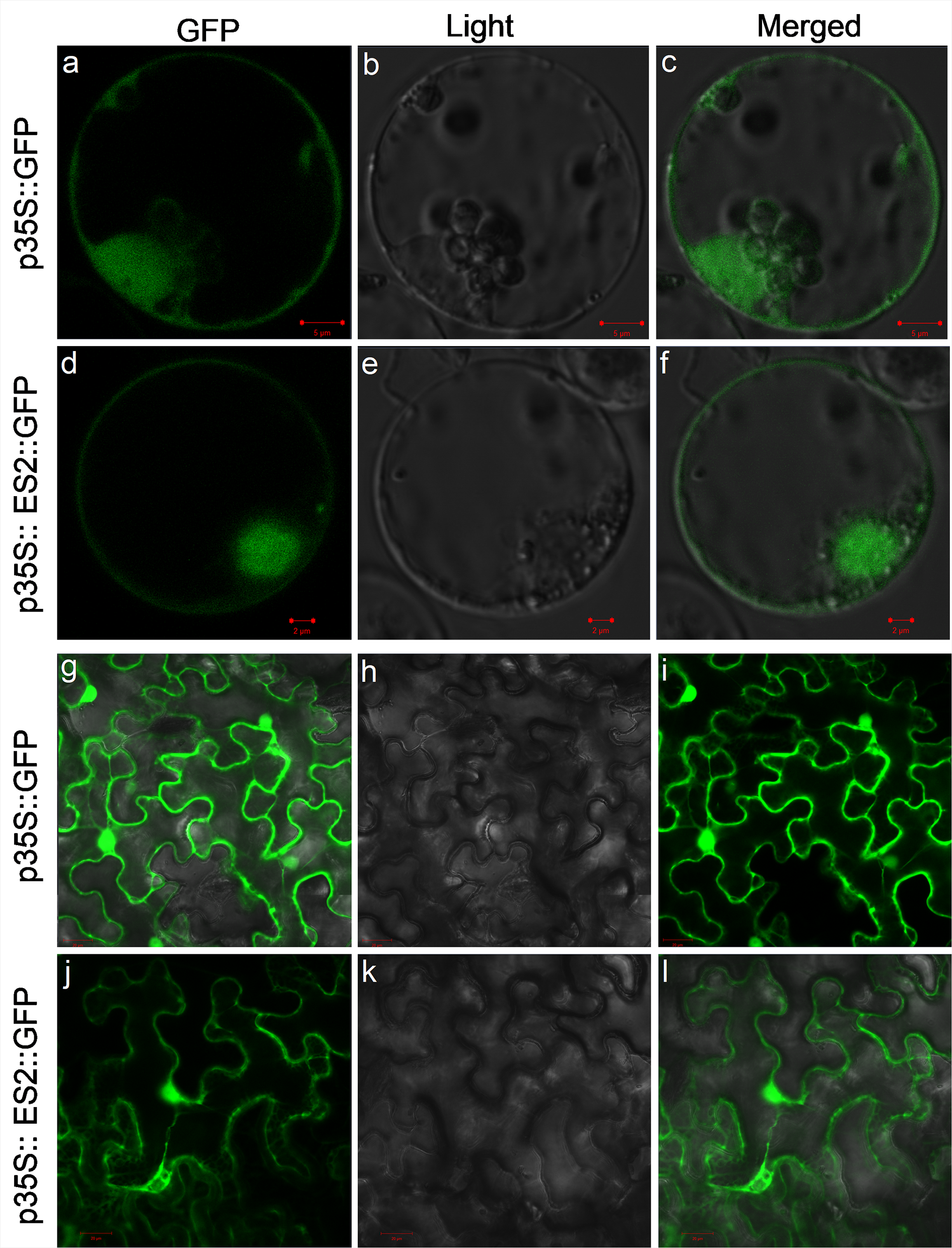

Supplement: Supplementary file 8 — Additional file 8: Figure S4. ES2 was localized to nucleus and plasma membrane in rice protoplasts and tobacco leaf epidermal cells. (a-c) Rice protoplast transformed with p35S::GFP as a control. Scale bar = 5 μm; (d-f) Rice protoplast transformed with p35S::ES2::GFP. Scale bar = 2 μm; (g-i) Tobacco (N. benthamiana) leaf epidermal cells transformed with p35S::GFP as a control. Scale bar = 20 μm; (j-l) Tobacco (N. benthamiana) leaf epidermal cells transformed with p35S::ES2::GFP. Scale bar = 20 μm. [file 12870_2020_2610_MOESM8_ESM.tif]

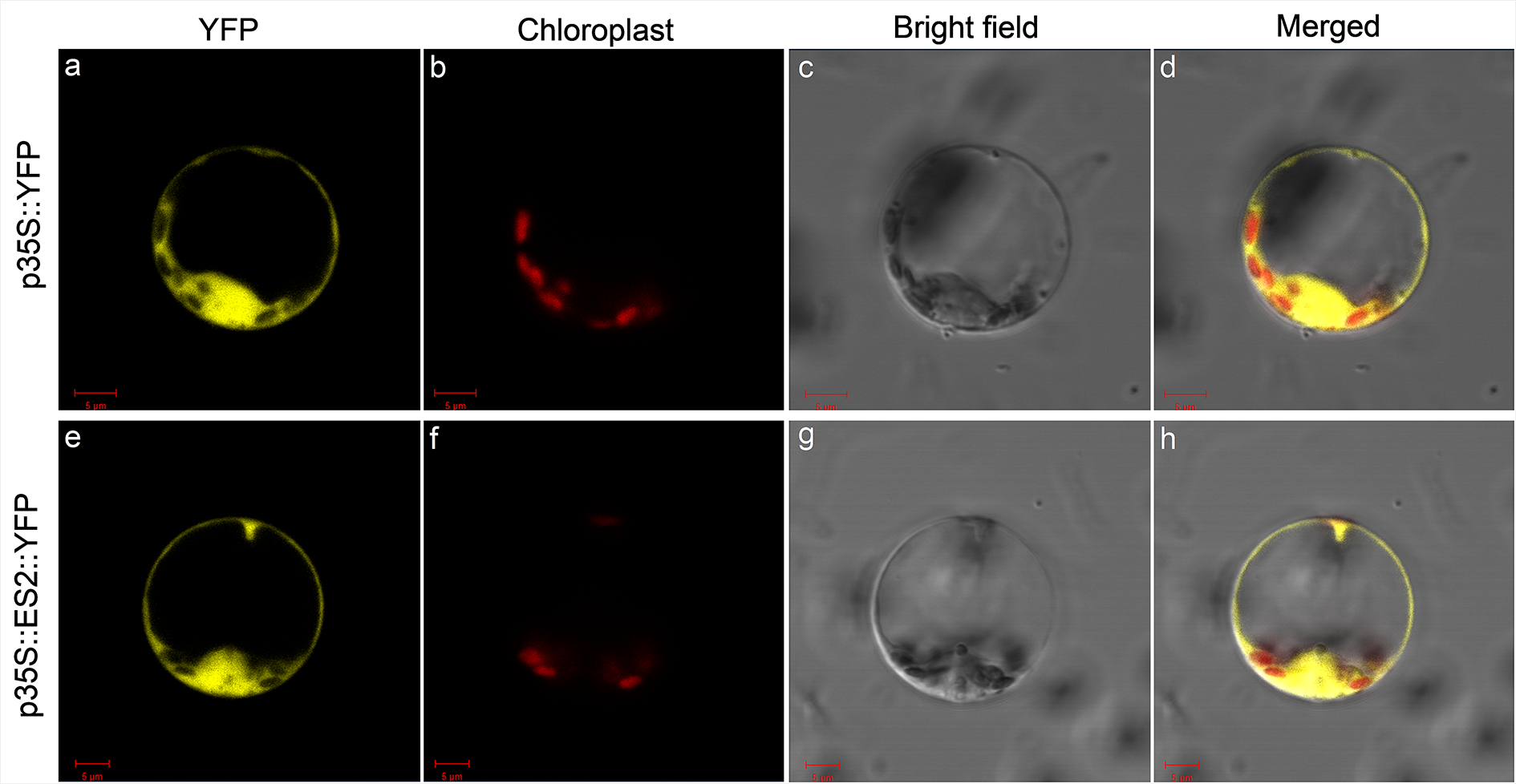

Supplement: Supplementary file 9 — Additional file 9: Figure S5. ES2 was localized to nucleus and plasma membrane in rice protoplasts transformed with p35S::YFP. (a-d) Rice protoplast transformed with p35S::YFP as a control. Scale bar = 5 μm; (e-h) Rice protoplast transformed with p35S::ES2::YFP. Scale bar = 5 μm. [file 12870_2020_2610_MOESM9_ESM.tif]

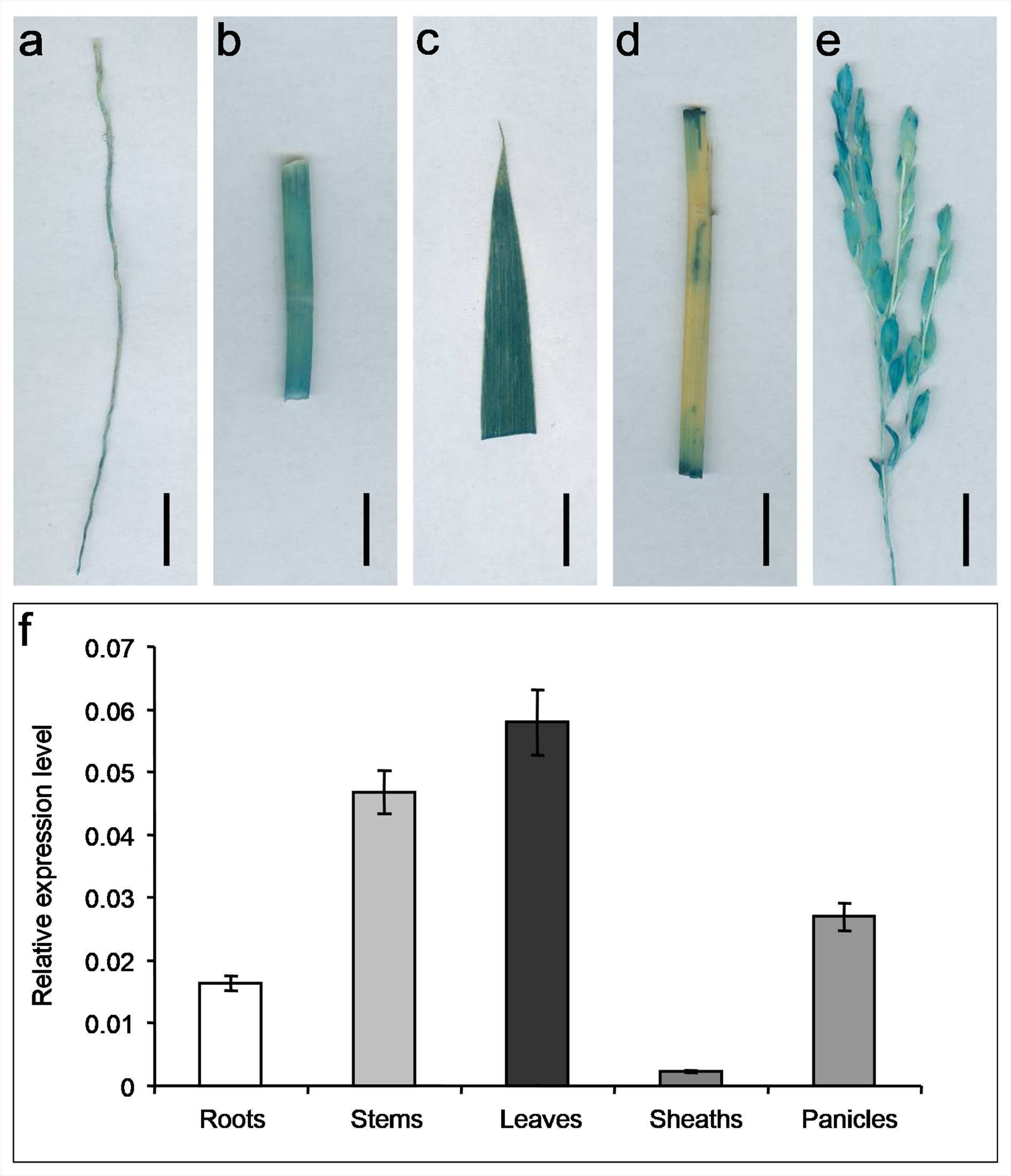

Supplement: Supplementary file 10 — Additional file 10: Figure S6. Tissue expression pattern of ES2. (a-e) GUS expression of transgenic rice with pES2::GUS at the heading stage. (a) Root. (b) Stem. (c) Leaf. (d) Sheath. (e) Panicle. Scale bar = 2 cm. (f) Relative expression levels of ES2 in various tissues revealed by qRT-PCR using Histone as the reference gene. Mean ± SD, n = 3. [file 12870_2020_2610_MOESM10_ESM.tif]

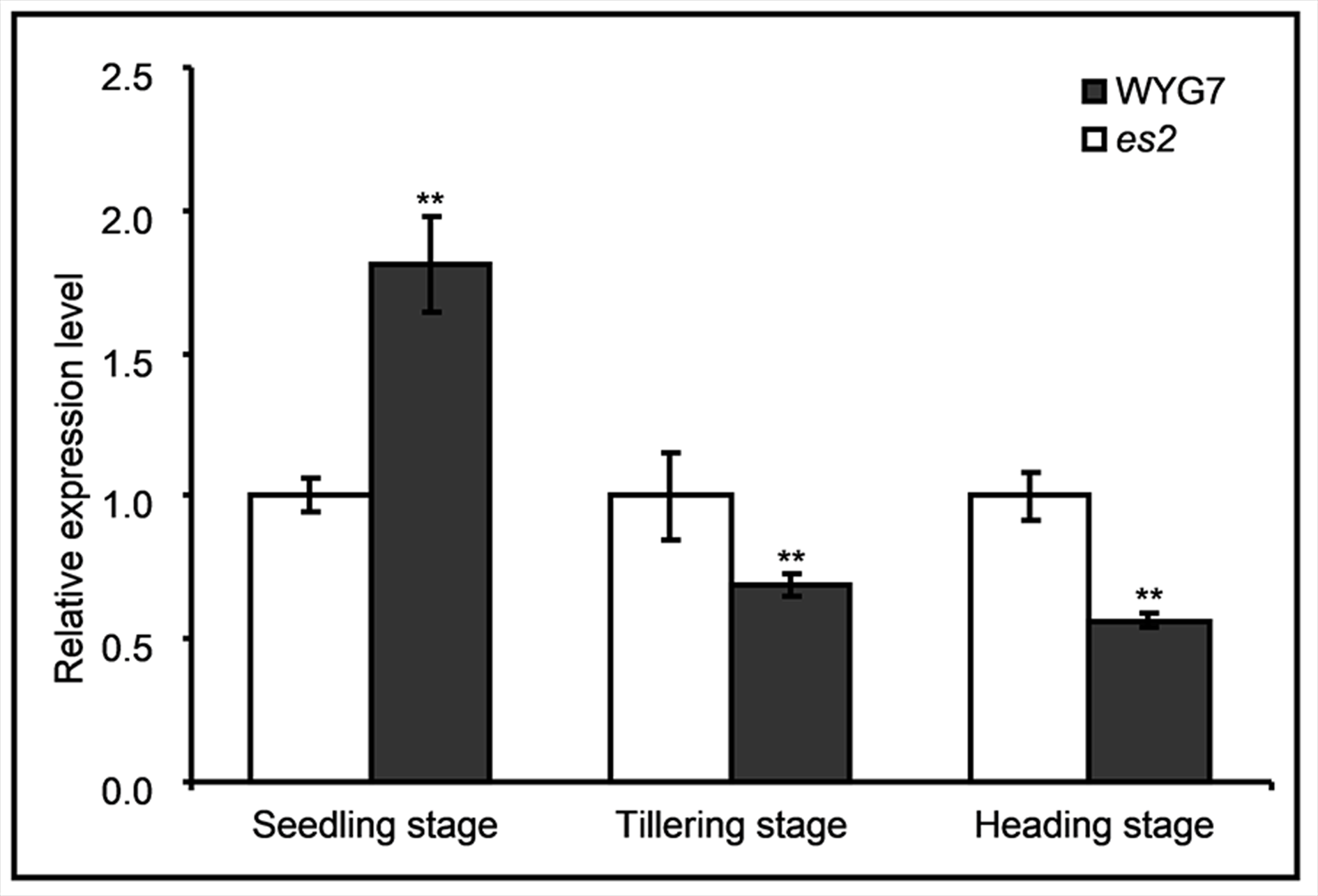

Supplement: Supplementary file 11 — Additional file 11: Figure S7. Expression levels of OsIPK2 in leaves from the wild type (WYG7) and the es2 mutants at seedling, tillering and heading stages. Histone gene was used as the reference. Mean ± SD, n = 3. * significance at P < 5%, ** extremely significance at P < 1% (Student’s t-test). [file 12870_2020_2610_MOESM11_ESM.tif]

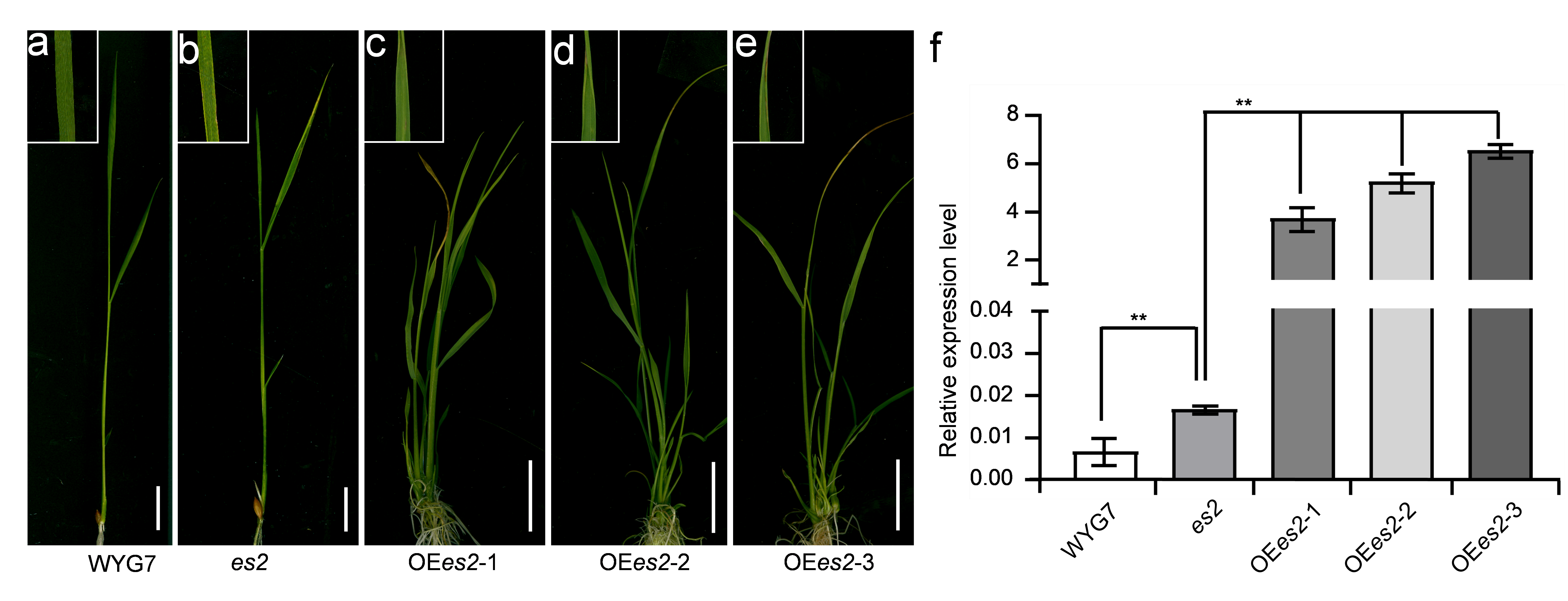

Supplement: Supplementary file 12 — Additional file 12: Figure S8. Leaf phenotype and relative expression level of OsIPK2 in seedlings of WYG7, es2 and overexpression lines OEes2–1, OEes2–2, and OEes2–3. (a) WYG7, (b) es2, (c) OEes2–1, (d) OEes2–2, (e) OEes2–3. Scale bar = 2 cm. (f) The relative expression level of OsIPK2 in WYG7, es2 and overexpression lines OEes2–1, OEes2–2, and OEes2–3. Mean ± SD, n = 3. ** significance at P < 1% (Student’s t-test). [file 12870_2020_2610_MOESM12_ESM.tif]

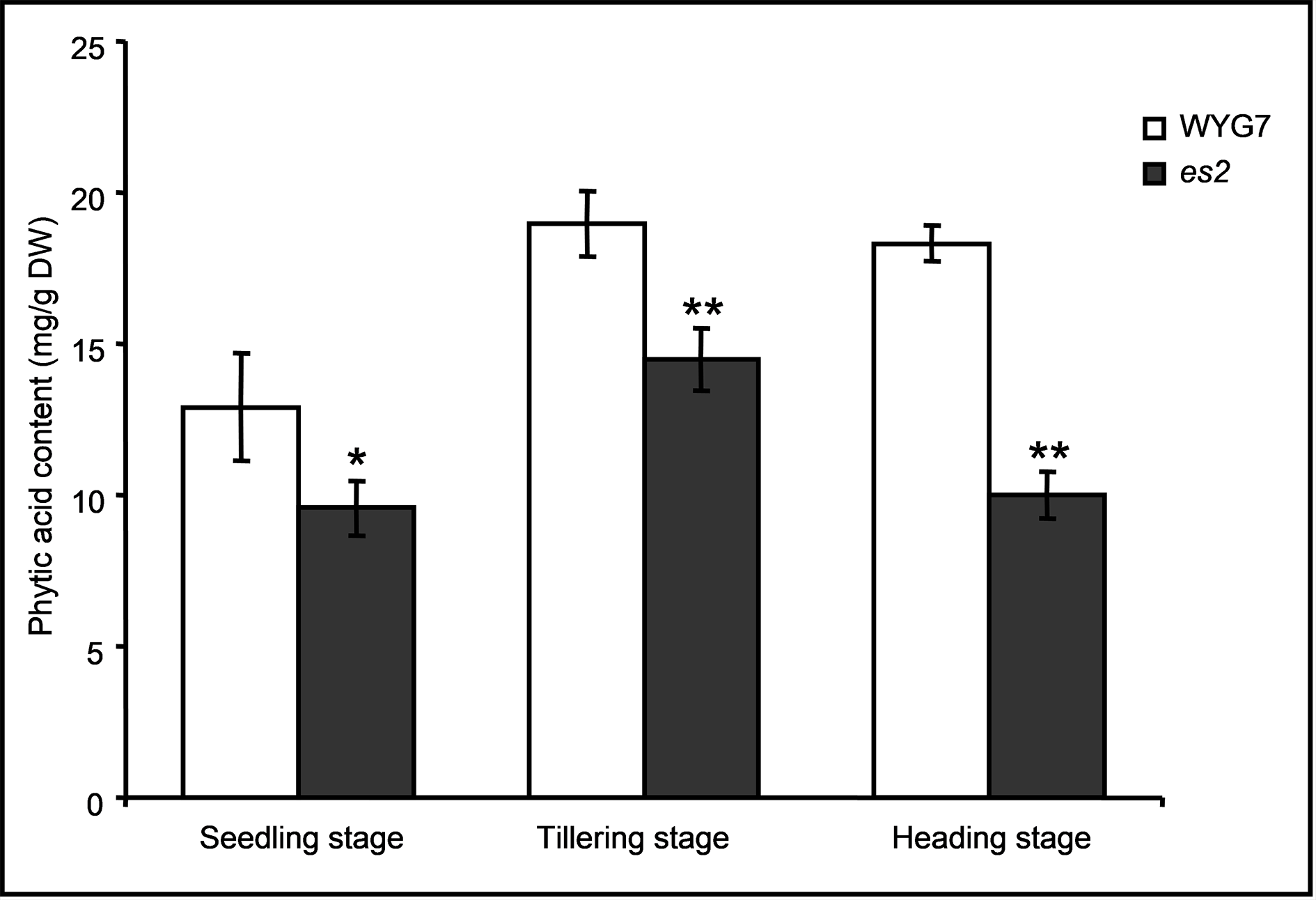

Supplement: Supplementary file 13 — Additional file 13: Figure S9. Determination of phytic acid content in leaves from the wild-type (WYG7) and the es2 mutants at seedling, tillering and heading stages. Mean ± SD, n = 3. * significance at P < 5%, ** significance at P < 1% (Student’s t-test). [file 12870_2020_2610_MOESM13_ESM.tif]
